# Bridging Two Worlds: Structural and Pharmacological Aspects of Natural Triterpenoid Dimers: Pristimerin-Pristimerin-Type Dimers

**DOI:** 10.3390/molecules31091386

**Published:** 2026-04-23

**Authors:** Andrzej Günther, Barbara Bednarczyk-Cwynar

**Affiliations:** 1 Department of Organic Chemistry, Faculty of Pharmacy, Poznan University of Medical Sciences, Collegium Pharmaceuticum 2 (CP.2), Rokietnicka Str. 3, 60-806 Poznan, Poland; bcwynar@ump.edu.pl; 2 Center of Innovative Pharmaceutical Technology (CITF), Rokietnicka Str. 3, 60-806 Poznan, Poland

**Keywords:** natural dimers, quinone methide triterpenoids, pristimerin derivatives, *Celastraceae* metabolites, biomimetic synthesis, atropisomerism

## Abstract

This review summarizes current knowledge on naturally occurring pristimerin-pristimerin triterpenoid dimers, a rare and structurally diverse class of secondary metabolites reported mainly from *Celastraceae* species. Known dimers are compiled with emphasis on botanical sources and key architectural features, including the variety of interunit linkages, regio- and stereochemical diversity, and distinct isomeric forms (including atropisomerism). Major advances in structure elucidation and structural revisions are discussed, highlighting the role of modern spectroscopic tools—particularly 2D NMR methods and chiroptical techniques—in resolving connectivity and absolute configuration, and in correcting several earlier assignments. Proposed biosynthetic scenarios are outlined, focusing on the reactivity of the quinone-methide motif and its interconversion with 2,3-diketone forms, as well as (hetero) Diels-Alder-type processes; selected biomimetic studies are summarized as supportive evidence for these pathways. A critical overview of available biological data indicates that many pristimerin dimers display limited activity in common antimicrobial and cytotoxicity assays when compared with monomeric congeners, which may point to alternative ecological roles or storage/transport functions in planta. Finally, key knowledge gaps and future directions are identified, including improved isolation coverage, rigorous synthetic/biomimetic work, and broader pharmacological screening beyond standard panels.

## 1. Introduction

Natural products have long played a pivotal role in drug discovery, providing structurally diverse molecules that have inspired numerous approved drugs and clinical candidates [1]. Among these, triterpenes and their derivatives (triterpenoids) constitute one of the most abundant and pharmacologically versatile classes of secondary metabolites [2]. Triterpenes originate biosynthetically from the cyclization of squalene or 2,3-oxidosqualene, giving rise to a wide variety of four- and five-ring carbon skeletons [3]. To date, more than 200 distinct triterpene frameworks have been identified, with extensive post-cyclization modifications further expanding their structural diversity [4].

Triterpenes are widely distributed in higher plants and also occur in fungi and some marine organisms [5]. They accumulate in leaves, bark, fruits, and cuticular waxes, where they fulfill protective, defensive, and signaling functions [6,7,8]. Prominent representatives such as oleanolic acid, ursolic acid, betulinic acid, and lupeol have attracted considerable attention owing to their broad biological activity spectrum and generally favorable safety profiles [9,10,11]. Chemical modification of these scaffolds—through oxidation, esterification, acetylation, or other derivatization strategies—often leads to triterpenoids with improved physicochemical properties, including enhanced solubility, bioavailability, and biological potency [9,12,13,14].

Extensive studies have demonstrated that triterpenes and triterpenoids exhibit a wide range of pharmacological activities, including anti-inflammatory, hepatoprotective, antioxidant, antimicrobial, and anticancer effects (e.g., [14,15,16,17]). At the molecular level, these compounds modulate key signaling pathways involved in inflammation, proliferation, and apoptosis, such as NF-κB (Nuclear factor kappa-light-chain-enhancer of activated B cells; nuclear factor kappa B), MAPK (mitogen-activated protein kinase), PI3K/Akt (phosphatidylinositol 3-kinase/protein kinase B signaling pathway), JNK (c-Jun N-terminal kinase), and STAT3 (signal transducer and activator of transcription 3), ultimately leading to cell cycle arrest or programmed cell death (e.g., [18,19]). In particular, oleanolic acid and its semisynthetic derivatives have been extensively investigated for anticancer potential, with reported mechanisms including apoptosis induction, inhibition of angiogenesis, suppression of metastasis, and regulation of cellular redox homeostasis (e.g., [20,21,22]).

In recent years, increasing interest has been directed toward dimeric triterpenoids, in which two triterpene units are covalently linked via ether, ester, or carbon–carbon bridges. Such dimerization strategies have frequently resulted in enhanced biological activity compared with monomeric analogues [23,24,25,26]. Notably, oleanolic acid dimers linked at the C-17 position have demonstrated low-micromolar or sub-micromolar cytotoxicity against various human cancer cell lines, accompanied by improved antioxidant activity and favorable ADMET-related properties [24,25,26]. The dimeric approach enables the incorporation of two bioactive fragments within a single molecular entity, potentially leading to cooperative effects or novel mechanisms of action.

Among naturally occurring triterpenoid dimers, quinonemethide dimers (celastroloids) are the most extensively studied subclass. These compounds, primarily isolated from plants of the *Celastraceae* family, consist of two tingenone- or pristimerin-type units connected through ether bridges, typically involving the A or B rings of each monomer [27]. Variations in linker type, stereochemistry, oxidation state, and substitution pattern have been shown to significantly influence their physicochemical properties and biological activity.

Despite the abundance of monomeric triterpenes and growing evidence that dimerization can enhance biological performance, systematic structure-activity relationship (SAR) studies of triterpenoid dimers remain limited, particularly for scaffolds derived from widely available natural products such as oleanolic acid. In particular, the influence of linker nature and length, relative orientation of monomeric units, oxidation state, and functional group substitution on anticancer activity and selectivity has not yet been comprehensively explored.

The scope of the present review is deliberately focused on naturally occurring pristimerin-pristimerin-type dimers and on closely related studies that are directly relevant to their structural characterization, stereochemical assignment, biosynthetic considerations, and biomimetic synthesis. These compounds represent a well-defined subclass of quinone methide triterpenoid dimers derived from pristimerin-type monomeric precursors and typically isolated from members of the *Celastraceae* family. Because these metabolites share a common structural framework and are believed to arise through closely related biosynthetic processes—most commonly rationalized in terms of highly regio- and stereoselective cycloaddition events—they constitute a coherent group suitable for comparative discussion of structural features, proposed biosynthetic pathways, stereochemical relationships, and mechanistic aspects of their formation.

To preserve this structural and mechanistic coherence, the review is intentionally restricted to dimers composed of two pristimerin-type units or to studies that bear directly on this structural motif, including reports describing structural revisions, reassignment of stereochemistry, and biomimetic transformations that model the proposed biosynthetic dimerization processes. Particular attention is therefore given to investigations that clarify the architecture of these dimers, their atropisomeric relationships, and the mechanistic rationale for the remarkable regio- and stereoselectivity observed in both natural biosynthetic pathways and laboratory biomimetic reactions.

In contrast, several related but structurally broader classes of compounds have been deliberately excluded from the present survey. These include mixed dimers, formed by the coupling of pristimerin-type units with other quinone methide triterpenoids; non-pristimerin-based celastroloid dimers, derived from structurally distinct celastroloid monomers; as well as structurally divergent triterpene dimers that do not belong to the celastroloid/quinone-methide triterpenoid family. Although these compounds may exhibit certain superficial similarities or arise from related plant sources, their structural diversity and potentially different biosynthetic origins would substantially broaden the scope of the discussion and obscure the mechanistic and structural relationships that are central to the present analysis. For this reason, these excluded groups fall outside the defined scope of this review and will be addressed separately in future work devoted specifically to these broader categories of triterpenoid dimers.

Articles included in the search were published starting from 1997. The information about the PCTTs was obtained from SciFinder, Scopus, and Web of Science, using as key search terms: *natural dimers*, *quinone methide triterpenoids*, *pristimerin derivatives*, *Celastraceae metabolites*. Mixed dimers, non-pristimerin-based celastroloids, and structurally distinct non-celastroloid triterpene dimers were excluded from the present review in order to maintain structural and mechanistic coherence.

## 2. Structural Diversity and Classification of Natural Triterpene Dimers

Among naturally occurring triterpene dimers, quinonemethide triterpenoid dimers represent the most abundant and structurally diverse group. These compounds, commonly known as *celastroloids*, were first defined by Brüning and Wagner as a distinct subclass of dimeric triterpenoids predominantly occurring in plant species of the *Celastraceae* family [27].

From a structural perspective, celastroloids consist of two quinonemethide triterpenoid moieties covalently linked by one or two ether bridges. These connections typically involve atoms located within ring A or ring B of one triterpenoid unit and ring A of the second unit, giving rise to a wide range of structural variants [27].

**Figure 1:**
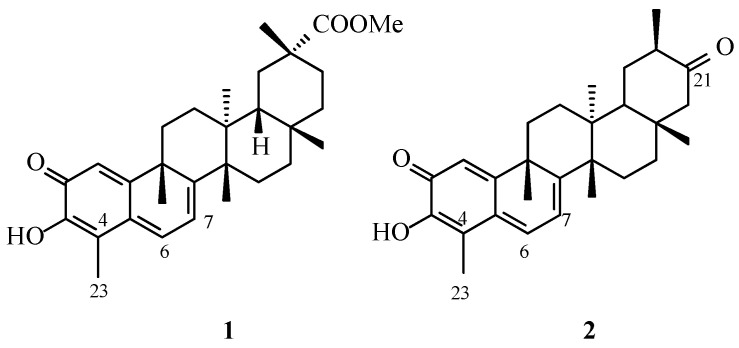
Structures of pristimerin (**1**) and tingenone (**2**).

The celastroloid class of natural dimeric triterpenoids comprises several principal structural groups, including xuxuarines, cangorosins, and scutionins, whose nomenclature reflects the plant species from which they were originally isolated. Despite their diversity, these compounds share a common architectural framework consisting of two triterpenoid subunits, most frequently derived from pristimerin or tingenone (**1** and **2**, Figure 1).

The primary distinction between pristimerin- and tingenone-type units does not lie in a different positional placement of the oxygenated functionality itself, but rather in the oxidation/substitution pattern around ring A and the resulting electronic character of the monomeric unit. These features determine the sites available for ether-bridge formation and influence the reactivity of the coupling partners. Accordingly, naturally occurring celastroloids may be constructed from two pristimerin-type units, two tingenone-type units, or a combination of both.

Furthermore, many naturally occurring triterpene dimers exist as dehydrogenated, aromatic analogues of pristimerin or tingenone, thereby further expanding the structural and chemical diversity of this distinctive class of natural products.

In addition to the celastroloid subclasses described above, a number of naturally occurring triterpene dimers have been identified that cannot be assigned to any of these established categories. These compounds possess structural characteristics that clearly distinguish them from quinonemethide-based dimers such as xuxuarines, cangorosins, and scutionins. In contrast to celastroloids, these dimers frequently feature alternative modes of covalent linkage between the monomeric triterpene units, lack quinoid structural motifs, or contain additional functional groups that substantially alter both their chemical architecture and biological profiles.

Owing to their distinctive architectures and structural diversity, these non-celastroloid triterpene dimers constitute a separate and relatively underexplored class of natural products. Their biosynthetic pathways, bonding arrangements, and potential pharmacological properties differ markedly from those found in conventional quinonemethide dimers. For these reasons, this group of compounds will be addressed independently in a dedicated study, which will provide a detailed analysis of their structural features, natural occurrence, and biological significance.

## 3. *Rzedowskia tolantonguensis* Pristimerin-Pristimerin-Type Triterpene Dimers

**Figure 2:**
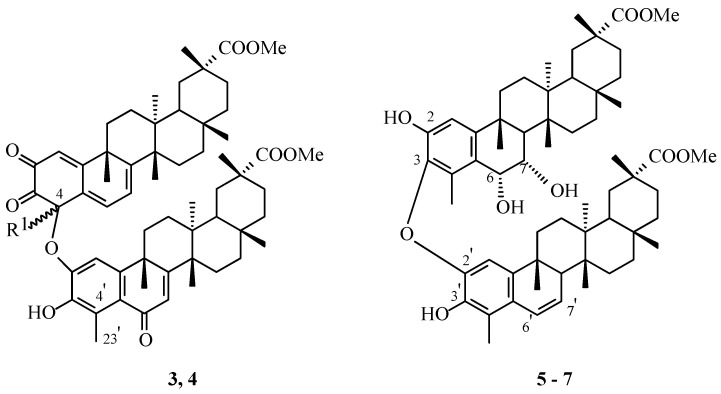
Structures of natural triterpene dimers **3**–**7**. **Legend**: **3**: “Compound **5**” by [28], R^1^ = α-CH_3_; **4**: “Compound **6**” by [28], R^1^ = β-CH_3_; **5**: cangorosin A, **6**: atropcangorosin A; **5** and **6**: atropisomers; **7**: 6′,7′-dihydroatropcangorosin A.

The initial discovery of pristimerin-pristimerin-type dimeric triterpenoids was reported by Gonzales and colleagues [28], who successfully isolated these compounds from the roots of *Rzedowskia tolantonguensis* (Medrano, *Celastraceae*). The structural elucidation of these dimers was accomplished through extensive Nuclear Magnetic Resonance (NMR) spectroscopic analysis, complemented by partial synthetic studies. In their experimental procedure, pristimerin was reacted with 2,3-dichloro-5,6-dicyanobenzoquinone (DDQ) in distilled dioxane at ambient temperature, employing a 1:1 molar ratio. This reaction yielded a mixture of four products, which were subsequently separated using preparative thin-layer chromatography. Among the resulting compounds, two predominant and more polar products, designated as “compound **5**” and “compound **6**” (Figure 2), exhibited spectroscopic characteristics identical to those of the corresponding naturally occurring products previously isolated from *R. tolantonguensis*.

Although comprehensive physical data were not reported, spectroscopic analysis indicated that both dimers (Figure 2) exhibited Proton Nuclear Magnetic Resonance (^1^H NMR) patterns comparable to those of pristimerin, with distinct features consistent with the formation of ether linkages between the triterpenoid units. The chemical shifts observed for the methyl groups at the C-23 and the C-23′ positions suggested the establishment of an ether bond connecting the C-4 carbon of one pristimerin molecule to the phenolic oxygen of the other. These structural inferences were corroborated by Carbon Nuclear Magnetic Resonance (^13^C NMR) data, which supported a molecular formula of C_60_H_78_O_9_. Additionally, Nuclear Overhauser Effect Spectroscopy (NOESY) experiments confirmed the spatial orientation of the substituents and established the α-configuration of the methyl group at the C-4.

These results constituted the first demonstration of naturally occurring pristimerin-pristimerin dimers, uncovering an atypical ether linkage motif within this class of quinonemethide triterpenoids. The work of Gonzales and colleagues consequently established a novel avenue in the study of *Celastraceae*-derived dimers, emphasizing both their structural intricacy and their potential biosynthetic significance.

## 4. *Maytenus ilicifolia* Pristimerin-Pristimerin-Type Triterpene Dimers—Preliminary Studies

Itokawa and colleagues [29] reported the isolation of four novel triterpenoid derivatives from a methanolic extract of *Maytenus ilicifolia* (Mart. ex Reissek, *Celastraceae*), all of which were confirmed to possess dimeric structures. Spectroscopic investigations indicated that three of these compounds—cangorosin A (**5**, Figure 2), atropcangorosin A (**6**, Figure 2), and 6′,7′-dihydroatropcangorosin A (**7**, Figure 2)—belong to the pristimerin-pristimerin-type dimer triterpenoids. All dimers were obtained as colorless, amorphous powders (Table 1).

Fast Atom Bombardment Mass Spectrometry (FAB-MS) analysis verified that cangorosin A (**5**, Figure 2) consists of two pristimerin-type units. Examination of the ^13^C NMR spectra revealed the presence of two aromatic rings bearing phenolic groups, connected via an ether linkage. One of the molecular fragments featured a 1,2-glycolic moiety at the C-6–C-7 positions, whereas the other retained an unsaturated C-6′=C-7′ bond. The observed NMR correlation data were fully consistent with the proposed structure of cangorosin A depicted in Figure 2 (compound **5**).

Atropcangorosin A (**6**, Figure 2), the second dimer isolated by Itokawa and co-workers [29], was identified as an atropisomer of cangorosin A (**5**, Figure 2). Both compounds share identical molecular frameworks, differing only in restricted rotation around a single bond, which allows them to be isolated as distinct entities. Atropcangorosin A was synthesized by heating a solution of dimer **3** (Figure 2) in N,N-dimethylformamide (DMF) at 150 °C. Compound **7**, 6′,7′-dihydroatropcangorosin A (Figure 2), exhibits a structure analogous to dimer **4** (Figure 2), except for the presence of a saturated bond between the C-6′ and the C-7′ atoms.

## 5. *Maytenus scutioides* Pristimerin-Pristimerin-Type Triterpene Dimers

**Figure 3:**
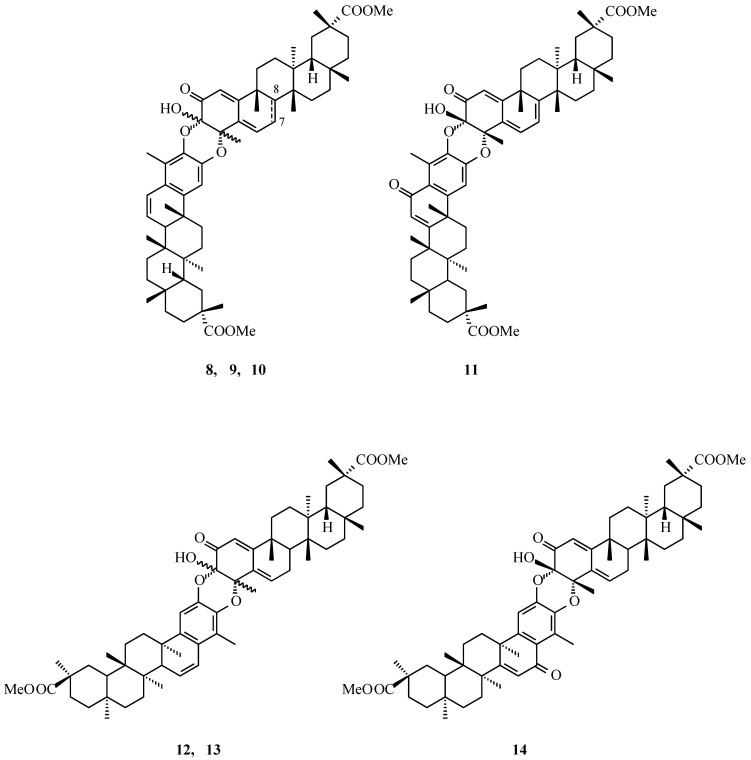
Structures of natural triterpene dimers **8**–**14**. **Legend**: **8**: scutionin αA; C-7,C-8: double bond; (3S,4S); 3β-OH, 4β-CH_3_. **9**: 7,8-dihydroscutionin αA; C-7,C-8: single bond; (3S,4S); 3β-OH, 4β-CH_3_. **10**: 7,8-dihydroscutionin βA; C-7,C-8: single bond; (3R,4R); 3α-OH, 4α-CH_3_. **11**: scutidin αA; **12**: 7,8-dihydroscutionin αB; (3S,4S); 3β-OH, 4β-CH_3_. **13**: 7,8-dihydroscutionin βB; (3R,4R); 3α-OH, 4α-CH_3_. **14**: 7,8-dihydroscutidin αB.

Gonzales and co-workers isolated eight novel triterpene dimers from the hexane-ether extract of *Maytenus scutioides* (Lourteig and O’Donnell, *Celastraceae*) roots, alongside several monomeric triterpenoids such as pristimerin and tingenone [30]. The structures of dimers **8**–**14** (Figure 3) were elucidated using standard NMR techniques in combination with circular dichroism (CD) analysis. Seven of these dimers were determined to be pristimerin-pristimerin-type compounds or derivatives exhibiting a similar structural arrangement.

Shirota and co-workers [31] proposed that the formation of these dimers may proceed through a hetero-Diels-Alder reaction. This biosynthetic hypothesis was further supported by a synthetic model: Gonzalez and colleagues [30] successfully generated the dimer by reacting an ortho-quinone with pristimerin, thereby demonstrating that the resulting product is structurally consistent with the naturally occurring dimers **8**–**14** (Figure 3).

The molecular formula of dimer **8** (Figure 3, scutionin αA, C_60_H_80_O_8_) was established based on FAB-MS and ^13^C NMR data and further corroborated by Infrared (IR) spectroscopy. The IR spectrum displayed absorption bands corresponding to a hydroxyl, carboxyl, and carbonyl groups (ν 3436 cm^−1^, 1731 cm^−1^, and ν 1678 cm^−1^, respectively). In the ^1^H NMR spectrum, eleven methyl signals were observed, including one aromatic methyl (δ 2.05 ppm) and two methoxy groups (δ 3.67 and 3.58 ppm). Three vinyl proton signals at δ 6.32, 6.06, and 5.92 ppm (Table 2), characteristic of a triterpene system in the quinone form, were assigned to the C-6, the C-1, and the C-7, respectively. The absence of analogous signals suggested that the compound comprises two pristimerin units, one in the quinone form and the other in the aromatic form. Analysis of ^13^C NMR, HMBC (Heteronuclear Multiple Bond Correlation), and HMQC (Heteronuclear Multiple-Quantum Correlation) data revealed that the quinone moiety possesses two oxidized quaternary carbons in ring A (the C-3, δ 91.81; the C-4, δ 78.73; Table 3), whereas the aromatic moiety contains a conjugated double bond between the C-6′ and the C-7′ (ring B) and two oxidized carbons in ring A (the C-2′ and the C-3′). NOE correlations between the C-3 hydroxyl group and the C-4 methyl group indicate that the two triterpene units are connected *via* two cis-oriented ether bridges.

**Figure 4:**
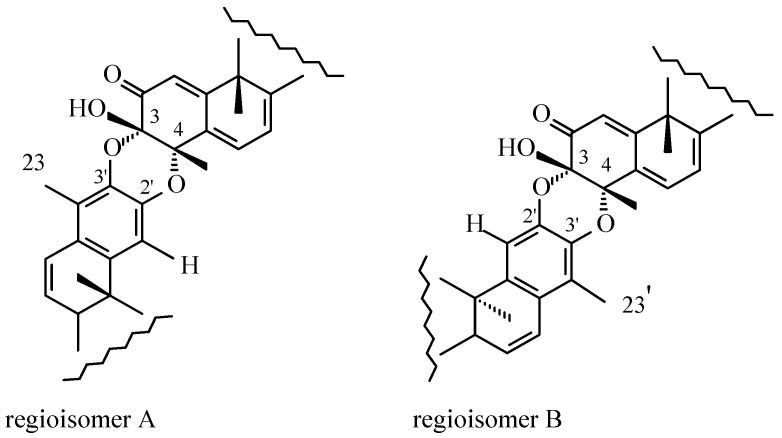
Regioisomers formed by a quinone and an aromatic form of pristimerin.

The quinoid form of pristimerin can combine with its aromatic counterpart to generate two possible regioisomers, designated as A and B (Figure 4). In regioisomer A, the C-3 carbon of the quinoid moiety is connected to the C-3′ carbon of the aromatic moiety, while the C-4 carbon of the quinoid unit forms a bond with the C-2′ of the aromatic unit, resulting in [3-*O*-3′] and [4-*O*-2′] linkages. In contrast, regioisomer B exhibits the reverse connectivity, with [3-*O*-2′] and [4-*O*-3′] linkages (Figure 4). NOE correlations observed between protons at the C-1′ and the C-6 positions confirmed that scutionin αA (**8**, Figure 3) corresponds to regioisomer A. This represents the first identification of a triterpene dimer bearing [3-*O*-3′] and [4-*O*-2′] linkages, with the absolute configuration at the C-3 and the C-4 assigned as 3S,4S.

**Table 2:** The representative chemical shifts for ^1^H NMR spectra of natural pristimerin-pristimerin-type triterpene dimers. **Legend**: * = atropcangorosin A according to [29]; ** = 6′,7′-dihydroatropcangorosin A (**7**) according to [29].

Compound Name	The Representative Chemical Shifts			Ref.
	δ [ppm] (*J*, [Hz])			
	C_1_-H; C_1′_-H	C_6_-H; C_6′_-H	C_7_-H; C_7′_-H	
“Compound **5**” (**3**)	6.08 (d, 1.3); 6.73 (s)	6.50 (dd, 6.8, 1.3); ---	6.08 (d, 6.8); 6.20 (s)	[28]
“Compound **6**” (**4**)	6.08 (d, 1.3); 6.80 (s)	6.23 (dd, 6.8, 1.3); ---	5.93 (d, 6.8); 6.24 (s)	
cangorosin A (**5**)	6.68 (s); 6.46 (s)	---; 6.66 (dd, 2.9, 10.0)	4.97 (dd, 2.9, 11.3); 5.89 (dd, 2.7, 10.0)	[29]
scutionin αA (**8**)	6.06 (d, 1.2); 6.70 (s)	6.32 (dd, 1.2, 6.3); 6.63 (dd, 2.7, 10.2)	5.92 (d, 6.3); 5.90 (dd, 2.5, 10.2)	[30]
7,8-dihydroscutionin αA (**9**)	5.94 (s); 6.67 (s)	6.34 (br/s); 6.64 (dd, 2.8, 9.9)	2.15 (m); 5.91 (dd, 2.6, 9.9)	
7,8-dihydroscutionin βA (**10**)	5.98 (s); 6.43 (s)	6.47 (br/s); 6.68 (dd, 2.2, 9.6)	2.13 (m); 5.91 (dd, 2.2, 9.6)	
scutidin αA (**11**)	6.09 (d, 1.4); 6.99 (s)	---; 6.30 (dd, 1.4, 6.5);	5.94 (d, 6.6); 6.21 (s)	
7,8-dihydroscutionin αB (**12**)	5.95 (s); 6.63 (s)	6.55 (br/s); 6.58 (dd, 1.6, 10.0)	2.15 (m); 5.88 (dd, 1.7, 10.0)	
7,8-dihydroscutionin βB (**13**)	5.95 (s); 6.50 (s)	6.23 (s); 6.66 (dd, 2.7, 10.0)	2.16 (m); 5.91 (dd, 2.2, 10.0)	
7,8-dihydroscutidin αB (**14**)	5.98 (s); 6.97 (s)	6.33 (s); ---	2.14 (m); 6.23 (s)	
cangorosin A, with revised structure (**17**)	6.68 (s); 6.46 (s)	4.88 (d, 2.9); 6.66 (dd, 2.9, 10.0)	4.97 (dd, 2.9, 11.3); 5.89 (dd, 2.7, 10.0)	[32]
isocangorosin A (**18**) *	6.68 (s); 6.56 (s)	4.87 (d, 3.0); 6.66 (dd, 2.9, 9.9)	5.06 (dd, 3.0, 11.2); 5.90 (dd, 2.6, 9.9)	
6′,7′-dihydroisocangorosin A (**19**) **	6.69 (s); 6.55 (s)	4.89 (d, 3.0 Hz); 2.73 (dd, 5.8, 16.9)	4.95 (dd, 3.0, 11.2); ---	
xuxuarine Eβ (**20**)	6.08 (d, 1.5); 6.74 (s)	6.52 (dd, 1.5, 7.0); ---	6.08 (d, 15.6); 6.21 (s)	[33]
xuxuarine Eβ (**20**)	6.08 (d, 1.6); 6.74 (s)	6.51 (dd, 6.9, 1.6); ---	6.08 (d, 6.9); 6.21 (s)	[34]
7,8-dihydroisoxuxuarine Eα (**21**)	5.97(s); ---	6.32 (br/s); ---	2.06 (m); 2.21 (m)	[35]
xuxuarine Eα (**22**)	6.09 (d, 1.2); 6.80 (s)	6.24 (dd, 1.2, 5.2); ---	5.95 (d, 5.2); 6.26 (s)	[36]
xuxuarine Eα (**22**)	6.09 (d, 1.6); 6.80 (s)	6.24 (dd, 1.6, 6.6); ---	5.94 (d, 6.6); 6.25 (s)	[34]
scutionin αB (**23**)	6.06 (d, 1.5); 6.49 (br/s)	6.21 (dd, 1.5, 6.5); 6.67 (dd, 2.9, 9.9)	5.94 (d, 6.7); 5.91 (dd, 2.4, 9.9)	[37]
6′,7′-dihydroscutionin αB (**24**)	6.05 (d, 1.4); 6.57 (s)	6.20 (dd, 1.5, 6.4); ---	5.92 (d, 6.5); ---	
6′β-methoxy-6′,7′-dihydroscutionin αB (**25**)	6.05 (d, 1.4); 6.55 (s)	6.18 (dd, 1.2, 6.4); 4.28 (br/s)	5.91 (d, 6.6); ---	
isoxuxuarine Eβ (**26**)	6.10 (d, 1.7); 6.96 (s)	6.56 (dd, 1.7, 7.0); ---	6.08 (d, 7.0); 6.21 (s)	[38]
7α-hydroxyisoxuxuarine Eα (**27**)	5.99 (d, 1.0); 6.98 (s)	6.15 (dd, 1.0, 3.1); ---	4.45 (ddd, 3.1, 9.2, 9.9); 6.22 (s)	
celastroline Aα (**31**)	6.07 (s); 6.53 (s)	6.25 (d, 6.0); ---	5.95 (d, 6.0); 6.19 (s)	[39]
celastroline Aβ (**32**)	6.06 (s); 6.73 (s)	6.52 (d, 6.6); ---	6.07 (d, 6.6); 6.20 (s)	
isocelastroline Aα (**33**)	6.06 (s); 6.99 (s)	6.23 (d, 6.0); ---	5.87 (d, 6.0); 6.18 (s)

**Table 3:** The representative chemical shifts for ^13^C NMR spectra of natural pristimerin-pristimerin-type triterpene dimers. **Legend**: **n.d.** = no data; * = atropcangorosin A according to [29]; ** = 6′,7′-dihydroatropcangorosin A (**7**) according to [29].

Compound Name	The Representative Chemical Shifts										Ref.
	δ [ppm]										
	C-1; C-1′	C-2; C-2′	C-3; C-3′	C-4; C-4′	C-5; C-5′	C-6; C-6′	C-7; C-7′	C-8; C-8′	C-9; C-9′	C-10, C-10′	
“Compound **5**” (**3**)	110.8; 119.9	179.2; 173.4	171.3; 145.3	91.2; 124.0	128.5; 132.0	129.0; 189.6	117.4; 126.3	164.5; 151.3	39.8; 44.0	137.7; 151.3	[28]
“Compound **6**” (**4**)	110.3; 115.3	188.0; 174.0	171.5; 144.7	92.1; 124.0	127.7; 130.1	126.7; 189.0	116.2; 126.2	161.4; 150.5	38.2; 41.9	137.7; 151.0	
cangorosin A (**5**)	108.30; 107.97	144.41; 141.60	140.68; 139.07	123.17; 122.01	124.69; 124.13	n.d.	n.d.	n.d.	n.d.	143.66; 143.76	[29]
scutionin αA (**8**)	115.60; 108.03	191.12; 140.04	91.81; 137.61	78.73; 122.42	130.70; 125.02	126.34; 124.01	116.30; 129.12	160.51; 45.51	41.62; 38.23	173.71; 143.72	[30]
7,8-dihydroscutionin αA (**9**)	112.83; 108.10	192.27; 140.10	91.37; 137.50	78.86; 122.49	134.12; 124.96	134.01; 124.01	29.68; 129.20	41.65; 45.55	37.36; 37.49	170.42; 143.71	
7,8-dihydroscutionin βA (**10**)	n.d.	n.d.	n.d.	n.d.	n.d.	n.d.	n.d.	n.d.	n.d.	n.d.	
scutidin αA (**11**)	115.72; 110.42	190.32; 144.35	91.68; 138.24	79.25; 129.20	130.39; 123.22	126.24; 187.20	116.01; 126.15	161.16; 170.95	41.70; 40.01	173.39; 151.66	
7,8-dihydroscutionin αB (**12**)	112.78; 108.13	192.14; 139.78	91.02; 137.39	77.20; 122.14	135.51; 125.26	136.84; 123.99	30.04; 129.17	43.13; 45.39	38.76; 37.10	170.23; 143.72	
7,8-dihydroscutionin βB (**13**)	112.73; 108.81	191.17; 140.31	91.28; 136.53	79.22; 121.29	133.91; 125.97	133.76 124.01	30.56; 129.40	41.80; 45.46	37.39; 37.39	170.38; 142.72	
7,8-dihydroscutidin αB (**14**)	112.70; 110.14	191.20; 144.17	90.95; 137.94	79.15; 129.03	133.72; 122.94	133.72; 186.85	29.09; 125.95	41.29; 170.58	39.81; 39.75	169.81; 151.41	
cangorosin A (**17**), with revised structure	108.30; 107.97	144.41; 141.60	140.68; 139.07	123.17; 122.01	124.69; 124.13	71.49; 124.29	74.49; 128.56	45.07; 45.81	40.32; 37.52	143.66; 143.76	[32]
isocangorosin A (**18**) *	108.18; 108.18	144.44; 142.08	140.59; 138.04	122.96; 121.05	124.68; 125.69	71.34; 124.41	74.25; 128.86	44.96; 45.93	40.28; 37.62	143.91; 141.93	
6′,7′-dihydroisocangorosin A (**19**) **	108.21; 109.48	144.29′ 140.12	140.61; 138.86	123.21; 125.60	124.59; 123.63	71.37; 28.05	73.69; 18.47	44.71; 44.15	40.25; 36.86	143.67; 145.07	
xuxuarine Eβ (**20**)	114.7; 110.6	189.4; 145.2	91.0; 137.5	77.3; 128.3	131.8; 123.8	128.8; 187.4	117.2; 126.1	164.4; 171.2	43.9; 40.0	173.2; 151.1	[33]
xuxuarine Eβ (**20**)	114.64; 110.62	187.35; 145.13	91.01; 137.51	76.89; 128.30	131.77; 123.79	128.81; 189.42	117.15; 126.12	164.36; 171.12	43.87; 39.98	173.23; 151.09	[34]
7,8-dihydroisoxuxuarine Eα (**21**)	113.0; 110.5	191.5 144.5	91.3; 138.3	79.5; 129.4	134.1; 123.3	134.1; 187.2	124.2; 126.3	41.6 171.0	37.4; 40.1	170.2; 151.8	[35]
xuxuarine Eα (**22**)	115.1; 111.3	190.2; 144.5	91.9 137.5	79.2; 127.5	129.9; 124.4	126.7; 187.8	116.0; 126.0	161.3; 171.6	44.6; 41.2	174.1; 150.4	[36]
xuxuarine Eα (**22**)	115.17; 111.37	187.89; 144.57	91.98; 137.55	79.31; 127.59	129.87; 124.44	126.77; 190.22	116.12; 126.11	161.39; 171.67	41.93; 39.94	174.15; 150.45	[34]
scutionin αB (**23**)	115.4; 108.8	191.0; 140.8	91.8; 136.5	79.1; 121.3	130.6; 126.0	126.1; 124.0	116.2; 129.4	160.8; 45.5	41.8; 37.4	173.8; 142.8	[37]
6′,7′-dihydroscutionin αB (**24**)	115.4; 111.4	191.2; 139.1	91.7; 136.2	79.9; 122.9	130.8; 127.9	126.1; 26.4	116.2; 18.5	160.6; 43.9	41.7; 36.8	173.8; 144.4	
6′β-methoxy-6′,7′-dihydroscutionin αB (**25**)	115.5; 110.4	191.0; 141.3	91.8; 137.0	79.0; 125.5	130.8; 127.2	125.9; 75.2	116.1; 21.8	160.6; 38.5	41.7; 37.6	173.5; 144.7	
isoxuxuarine Eβ (**26**)	114.9; 110.7	189.6; 144.2	90.7; 138.5	77.2; 128.2	132.1; 124.0	128.7; 187.8	116.9; 126.2	164.5; 171.5	44.1; 39.9	173.3; 151.1	[38]
7α-hydroxyisoxuxuarine Eα (**27**)	114.0; 110.6	191.2; 144.2	91.3; 138.0	79.1; 129.3	134.7; 123.5	135.9; 187.2	68.5; 126.2	51.7; 171.0	41.1; 40.2	168.7; 151.9	
celastroline Aα (**31**)	116.0; 111.2	187.8; 144.4	92.2; 137.5	79.1; 127.2	130.3; 124.4	126.6; 192.0	116.0; 126.0	161.8; 171.4	41.9; 40.3	174.4; 150.1	[39]
celastroline Aβ (**32**)	114.8; 110.8	187.3; 145.0	90.8; 137.4	76.9; 128.3	131.7; 123.6	128.8; 189.9	117.3; 126.1	164.4; 171.0	43.8; 40.0	173.4; 151.1	
isocelastroline Aα (**33**)	115.9; 110.4	190.8; 144.7	92.0; 138.2	79.4; 129.5	130.5; 123.0	125.9; 187.5	116.2; 126.0	161.2; 171.4	41.6; 40.0	173.4; 151.9	

Dimers **9** and **10** (Figure 3) both possess the molecular formula C_60_H_82_O_8_. In comparison to compound **8** (Figure 3), their ^1^H NMR spectra lack the signal at δ 5.92 ppm (Table 2) corresponding to the proton at the C-6 position of the quinone moiety, and instead display new signals at δ 6.43 and 6.47 ppm (Table 3), consistent with the saturation of the C-7–C-8 double bond. Both dimers are connected through [3-*O*-3′] and [4-*O*-2′] ether linkages, with the absolute configurations at the C-3 and the C-4 atoms assigned as 3S,4S for dimer **9** and 3R,4R for dimer **10**. These compounds were designated as 7,8-dihydroscutionin αA and 7,8-dihydroscutionin βA, respectively.

Dimer **11** (Figure 3) was obtained as a yellow amorphous solid. Spectroscopic analysis indicated that one triterpene unit corresponds to the quinoid form of pristimerin, similar to scutionin αA (**8**, Figure 3), while the second unit was aromatic, containing a carbonyl group in ring B and a conjugated double bond between the C-7′ and the C-8′ atoms. Diagnostic ^1^H NMR signals were observed at δ 6.21 ppm (Table 2; the C-7′, α,β-unsaturated ketone) and δ 6.99 ppm (Table 2; the C-1′, aromatic proton). The two units are linked via [3-*O*-3′] and [4-*O*-2′] ether bonds, and the absolute configuration at the C-3 and the C-4 was 3S,4S.

Compounds **12** and **13** were identified as a pair of diastereomeric dimers sharing the molecular formula C_60_H_82_O_8_, closely related to dimers **9** and **10** shown in Figure 3. Their NMR spectroscopic data exhibited a high degree of similarity, with the principal distinction arising from the nature of the ether bridges. In compound **12**, the linkages were assigned as [3-*O*-2′], whereas compound **13** displayed [4-*O*-3′] connectivity (Figure 3). Stereochemical analysis established the absolute configurations of the C-3 and the C-4 as 3S,4S for compound **12** and 3R,4R for compound **13**. Accordingly, these compounds were designated as 7,8-dihydroscutionin αB (**12**) and 7,8-dihydroscutionin βB (**13**), respectively.

The final pristimerin-derived dimer, compound **14** (Figure 3), was characterized as 7,8-dihydroscutidin αB and showed close structural resemblance to scutidin αA (**11**, Figure 3). However, its ^1^H NMR spectrum lacked the characteristic doublet at δ 5.94 ppm corresponding to a proton at the C-7 position, consistent with saturation of the C-7–C-8 double bond. Analysis of the Nuclear Overhauser Effect (NOE) correlations supported the presence of [3-*O*-2′] and [4-*O*-3′] ether linkages, while the absolute configuration at the C-3 and the C-4 was determined to be 3S,4S.

In addition to the pristimerin-derived dimers described above, Gonzales et al. reported the isolation of one further dimeric metabolite whose structural framework differs from the pristimerin-pristimerin type; its structural elucidation will be addressed elsewhere in the present work.

Dimers **8**–**14** (Figure 3) were systematically evaluated for their biological activity, including antibacterial and cytotoxic effects. Antimicrobial assays were conducted against a panel of nine bacterial strains: *Staphylococcus aureus* ATCC 6538, *S. epidermidis* CECT 232, *S. saprophyticus* CECT 235, *Bacillus subtilis* CECT 39, *B. pumilus* CECT 29, *Escherichia coli* CECT 99, *Proteus mirabilis* CECT 170, *Salmonella typhimurium* UBC 2, and *Pseudomonas aeruginosa* AK 958. Evaluation of cytotoxic potential was performed using human cervical carcinoma (HeLa) and human laryngeal carcinoma (Hep-2) cell lines. Under the conditions employed, none of the tested dimers (**8**–**14**, Figure 3) exhibited significant antibacterial or cytotoxic activity (Table 4).

**Figure 5:**
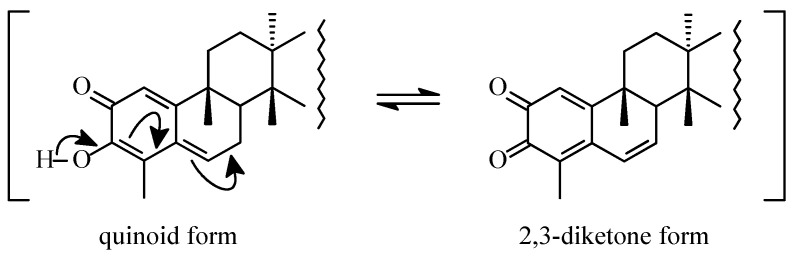
Equilibrium state of a quinone form of pristimerin with its 2,3-diketone form.

According to the biosynthetic hypothesis advanced by Shi [31], the formation of triterpene dimers proceeds through a Diels-Alder cycloaddition as a central step. In this proposal, the quinoid form of the monomer exists in equilibrium with a corresponding 2,3-diketone tautomer (Figure 5), either of which may function as a reactive species during dimerization.

The pronounced regio- and stereoselectivity observed in the dimerization of quinonemethide triterpenoids can be rationalized by considering both the intrinsic structural features of the reacting monomers and the nature of the proposed hetero-Diels-Alder-type cycloaddition mechanism. In the biosynthetic pathway, the quinoid form of the triterpene exists in equilibrium with its corresponding 2,3-diketone tautomer, either of which may serve as a reactive diene or dienophile during the cycloaddition process.

The rigid pentacyclic framework of these molecules strongly restricts conformational flexibility, preorganizing the π-systems in a defined spatial arrangement and favoring a limited number of energetically accessible transition states. Steric shielding by angular methyl groups and substituents on the A and B rings further directs the approach of the reacting partners, preferentially stabilizing endo-type transition states in which secondary orbital interactions between the conjugated π-systems can occur. As a result, specific orientations of the diene and dienophile become energetically favored, leading to highly selective formation of particular regioisomeric and stereoisomeric dimers. In biosynthesis, this selectivity may be further enhanced by enzyme-mediated preorganization within a catalytic pocket, potentially involving oxidases or Diels-Alderase-like proteins that orient the substrates in a defined geometry prior to bond formation.

Notably, comparable selectivity is often observed in biomimetic synthetic systems, indicating that the inherent steric and electronic properties of the quinonemethide scaffold play a dominant role in determining the preferred transition state. Consequently, both enzymatic control in planta and substrate-controlled cycloaddition in vitro contribute to the remarkably consistent regio- and stereochemical outcomes observed for these triterpene dimerization reactions.

**Figure 6:**
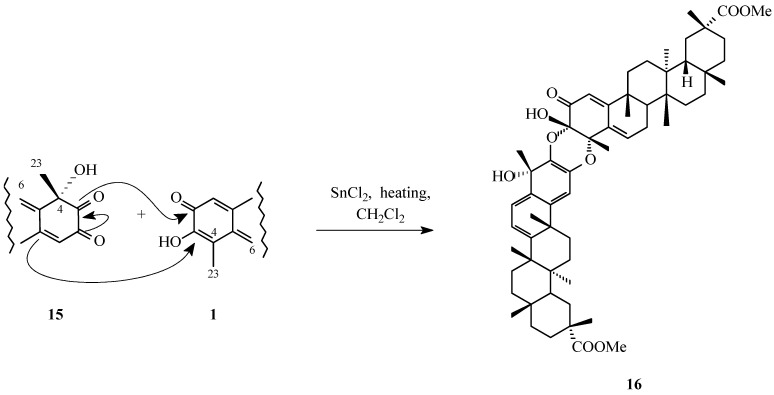
Biomimetic formation of triterpene dimer **16** from pristimerin (**1**) and 4α-hydroxypristimerin (**15**); the scheme illustrates a plausible cycloaddition-based coupling mode.

To experimentally examine this concept, Gonzáles et al. [30] carried out a biomimetic study in which pristimerin (**1**, Figure 1) was first oxidized with dimethyldioxirane (DMDO), affording 4α-hydroxypristimerin (**15**, Figure 3). This intermediate was subsequently allowed to react with pristimerin (**1**, Figure 1) under conditions favorable for a Diels-Alder reaction. The process yielded a series of regio- and stereochemically distinct dimeric products, including compound **16** (Figure 6), whose structures were established through detailed spectroscopic analysis.

## 6. *Maytenus ilicifolia* Pristimerin-Pristimerin-Type Triterpene Dimers—Advanced Structural Studies

As we presented earlier, in a study conducted by Itokawa and co-workers, four dimeric triterpenes were isolated from a methanolic extract of *Maytenus ilicifolia* (Mart. ex Reissek, *Celastraceae*) [29]. Detailed structural analyses confirmed the dimeric nature of all four metabolites, three of which were conclusively identified as pristimerin-pristimerin conjugates. Subsequent reinvestigation of these compounds, employing advanced NMR methodologies—particularly inverse-detection two-dimensional NMR techniques—led to a reassessment and refinement of the originally proposed structural assignments [32].

These findings provided additional support for the biogenetic pathway previously suggested by Shirota et al. [31] and, moreover, expanded the understanding of the synthetic potential and chemical reactivity associated with this class of triterpene dimers.

**Figure 7:**
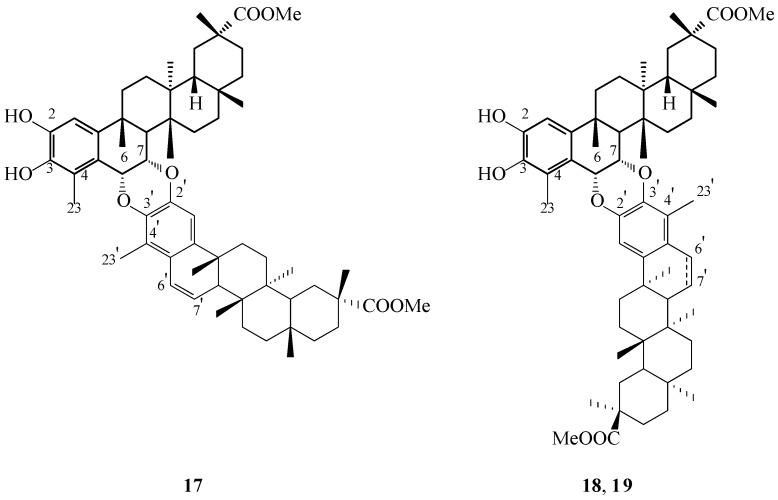
Structures of natural triterpene dimers **17**–**19**. **Legend**: **17**: cangorosin A, revised structure. **18**: isocangorosin A; 6′,7′: double bond. **19**: 6′,7′-dihydroatropcangorosin A; 6′,7′: single bond.

First of the identified pristimerin-pristimerin dimer, originally designated cangorosin A (**5**, Figure 2), was first proposed to consist of two aromatized pristimerin moieties linked through a single ether bond of the [3-*O*-2′] type. In this initial structural assignment, two hydroxyl substituents were placed in a cis relationship at the C-6 and the C-7 positions of one triterpene unit. Subsequent chemical derivatization with trichloroacetyl isocyanate, together with analysis of the resulting spectroscopic data, demonstrated instead that these hydroxyl groups were located at the C-2 and the C-3. Moreover, HMBC correlations obtained from a dimethylated derivative provided clear evidence for the presence of two cis-configured ether bridges, namely [6-*O*-3′] and [7-*O*-2′], thereby leading to a revised and definitive structural proposal (**17**, Figure 7).

A second related dimer, termed atropcangorosin A (**6**, Figure 2) by Itokawa and co-workers, was subsequently shown to comprise the same triterpene building blocks as cangorosin A (**17**, Figure 7) [29]. Detailed spectroscopic analyses and comparative studies performed by Shirota [32] established that atropcangorosin A represents an atropisomer of cangorosin A. As a consequence, the structure of compound **6** (Figure 2) was reassigned as depicted in Figure 7 (compound **18**) and the compound was accordingly renamed isocangorosin A.

The third dimeric triterpene isolated from *M. ilicifolia* by Itokawa [29], initially designated 6′,7′-dihydroatropcangorosin A (**7**, Figure 2), was subsequently reinterpreted by Shirota [32] as a hydrogenated analogue of dimer **18**. Detailed evaluation of two-dimensional NMR spectra established that saturation had occurred specifically at the C-6′ and the C-7′ positions. The revised molecular architecture of this compound is depicted in Figure 7 as compound **19**.

**Figure 8:**
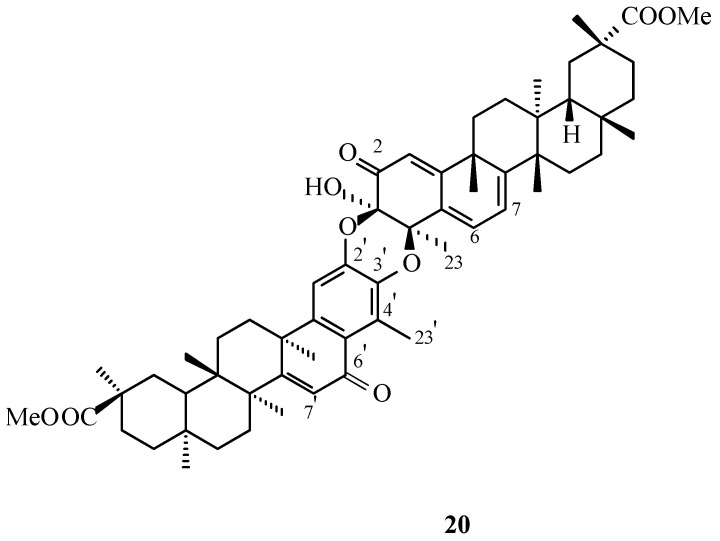
Structure of natural triterpene dimer **20**. **Legend**: **20**: xuxuarine Eβ.

In later investigations, Shirota and co-workers reported the isolation of four previously undescribed triterpene dimers from the dichloromethane fraction of a methanolic extract of *M. chuchuhuasca*, including one dimer composed of two pristimerin-derived units. This compound, designated xuxuarine Eβ (**20**, Figure 8), was assigned the molecular formula C_60_H_78_O_9_ based on HR-FAB-MS (High-Resolution Fast Atom Bombardment Mass Spectrometry). NMR spectroscopic analysis revealed that the molecule comprises one quinoid subunit (triterpene “a” unit, T_a_) and one aromatized subunit (triterpene “b” unit, T_b_). The observed chemical shifts for C-4 (δ 77.3) and C-23 (δ 24.6) were consistent with a β-oriented ether linkage at C-4 (Table 3). Structural elucidation further demonstrated that the two monomeric units are connected through cis-configured ether bridges of the [3-*O*-2′] and [4-*O*-3′] types (Figure 8). In addition, spectroscopic evidence confirmed the presence of two conjugated double bonds within the B ring of the T_a_ unit and a conjugated enone system in the B ring of the T_b_ unit [33].

Xuxuarine Eβ (**20**, Figure 8) is another example of a triterpene dimer composed of two pristimerin-type monomeric units. The structural diversity observed among such dimers, which arise through Diels-Alder reactions, is attributable to the capacity of a 2,3-diketone triterpene—capable of interconverting with its quinoid tautomer—to interact with a partner monomer from multiple faces and in various spatial orientations. These different approaches give rise to distinct geometric and stereochemical isomers, including α/β dimer pairs and dimer/isodimer variants.

## 7. *Maytenus chuchuhuasca* Pristimerin-Pristimerin-Type Triterpene Dimers

**Figure 9:**
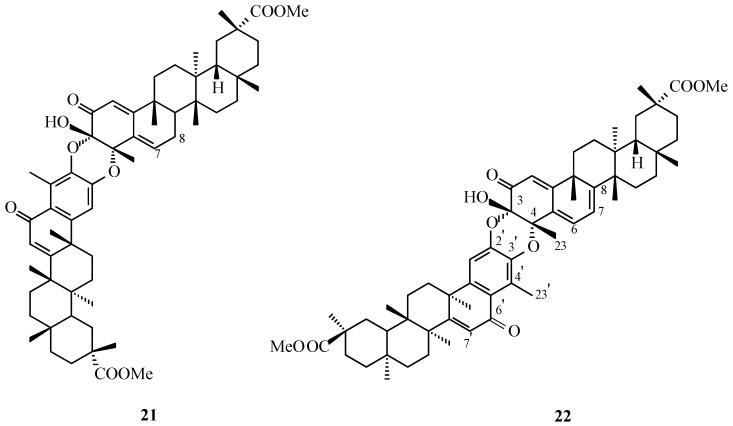
Structure of natural triterpene dimers **21** and **22**. **Legend**: **21**: 7,8-dihydroisoxuxuarine Eα. **22**: xuxuarine Eα.

In their continued investigation of *Maytenus* species, Shirota and colleagues examined the methylene chloride-soluble (CH_2_Cl_2_-soluble) fraction of a methanolic extract of *M. chuchuhuasca* (primary botanical name: *M. macrocarpa*, (Ruiz & Pav.) Briq., *Celastraceae*) [35]. Sequential purification using silica gel chromatography, followed by medium-pressure liquid chromatography and octadecylsilyl liquid chromatography, afforded five minor triterpene dimers. Among these, two were pristimerin-pristimerin dimers: scutidin αA (previously isolated as isoxuxuarine Eα (**11**, Figure 3) and 7,8-dihydroisoxuxuarine Eα (**21**, Figure 9).

Scutidin αA (compound **11**, Figure 3) was obtained as a yellow, amorphous solid (Table 1). High-resolution FAB-MS analysis revealed an [M+H]^+^ ion at *m*/*z* 943, consistent with the molecular formula C_60_H_78_O_9_. Infrared spectroscopy indicated the presence of a single free hydroxyl group (ν 3434 cm^−1^). NMR data confirmed that dimer **11** is composed of two pristimerin units, with the T_a_ moiety adopting a quinoid structure and the T_b_ moiety an aromatic structure, resembling the arrangement observed in xuxuarine Eβ (**20**, Figure 8).

The NOESY spectrum of the 3-*O*-methyl derivative provided further support for the stereochemical assignment. Key Nuclear Overhauser Effect (NOE) correlations were observed between the protons of the introduced methoxy group at the C-3 and the protons at the C-23 and the C-23′ atoms, confirming the cis-orientation of the 3,4-dioxy linkages. These correlations established that the two units are connected in an iso-type fashion via [3-*O*-3′] and [4-*O*-2′] ether bonds. Collectively, these spectroscopic data corroborate that scutidin αA (**11**) represents an iso-type analogue of xuxuarine Eα, as depicted in Figure 3.

The second dimer, designated as compound **21** (Figure 9) was isolated as a pale yellow, amorphous solid and exhibited an [M+H]^+^ ion at *m*/*z* 945, corresponding to the molecular formula C_60_H_80_O_9_. NMR analysis confirmed that the compound consists of two pristimerin units, with the T_a_ moiety in a quinoid form and the T_b_ moiety in an aromatic form, although notable differences distinguish it from compounds **11** (Figure 3) and **20** (Figure 8). The absence of the olefinic proton signals for the C-6 and the C-7 atoms, accompanied by the emergence of signals corresponding to the saturated C-7 and the C-8 atoms (Table 3), indicated hydrogenation at this position. Comprehensive analysis of HMQC, HMBC, NOESY, and CD data established that the dimer **21** (Figure 9) retains an iso-type connectivity pattern identical to that of compound **11** from Figure 3, featuring α-oriented cis [3-*O*-3′] and [4-*O*-2′] ether linkages. On this basis, compound **21** (Figure 9) was identified as 7,8-dihydroisoxuxuarine Eα.

## 8. *Maytenus blepharodes* Pristimerin-Pristimerin-Type Triterpene Dimers

Gonzales and colleagues [36] explored the chemical constituents of *Maytenus blepharodes* (Lundell, *Celastraceae*). During examination of the remaining extract fractions, the researchers isolated eight previously known triterpene dimers, all of which had been earlier identified in *M. scutioides* or *M. ilicifolia*. The compounds obtained included scutionin αA (**8**, Figure 3), 7,8-dihydroscutionin αA (**9**, Figure 3), scutidin αA (**11**, Figure 3), 7,8-dihydroscutionin αB (**12**, Figure 3), 7,8-dihydroscutionin βB (**13**, Figure 3), 7,8-dihydroscutidin αB (**14**, Figure 3) and these compounds were also reported in *M. scutioides* [30]). Additionally, cangorosin A (**17**, Figure 7), and 6′,7′-dihydroisocangorosin A (**19**, Figure 7), previously designated as 6′,7′-dihydroatropcangorosin A (**7**, Figure 2) according to Itokawa [29] were also isolated from *M. blepharodes* extract. In addition, a novel triterpene dimer was discovered and named xuxuarine Eα (**22**, Figure 9).

The newly identified triterpene dimer, designated compound **22** (Figure 9) was obtained as a pale yellow, amorphous solid (Table 1). FAB-MS analysis revealed an [M+H]^+^ ion at *m*/*z* 943, corresponding to the molecular formula C_60_H_78_O_9_, identical to that determined for xuxuarine Eβ (**20**, Figure 8). The IR spectrum of compound **22** (Figure 9) displayed characteristic absorption bands attributable to a free hydroxyl group (3444 cm^−1^), an ester carbonyl functionality (1731 cm^−1^), and a conjugated carbonyl group (1677 cm^−1^). NMR spectroscopic data demonstrated that compound **22** is composed of two pristimerin-derived units, one adopting a quinoid configuration (T_a_) and the other an aromatic configuration (T_b_). Two conjugated ketone functions were identified at the C-2 and the C-6′ positions (Table 3). The spectroscopic evidence further indicated a cis relationship across the 3,4-dioxy linkage. ROESY correlations established that the ether bridges correspond to [3-*O*-2′] and [4-*O*-3′] connectivities. Analysis of the circular dichroism (CD) spectrum allowed assignment of the absolute configuration of dimer **22** (Figure 9) as 3S,4S.

The collective spectroscopic and analytical evidence supported the conclusion that compound **22** (Figure 9) represents a constitutional isomer of both xuxuarine Eβ (**20**, Figure 8) and scutidin αA (**11**, Figure 3).

## 9. *Maytenus magellanica* Pristimerin-Pristimerin-Type Triterpene Dimers

As an extension of their investigations on *Maytenus blepharodes*, Gonzales and co-workers reported the isolation of a total of ten triterpene dimers—seven previously known and three novel—from extracts of *M. blepharodes* and *M. magellanica* ((Lam.) Hook.f., *Celastraceae*) [36]. Specifically, root bark (550 g) of *M. blepharodes* and roots (570 g) of *M. magellanica* were exhaustively extracted in a Soxhlet apparatus using an *n*-hexane-diethyl ether (1:1) solvent system, yielding 10.0 g and 13.5 g of dry extracts, respectively. These crude extracts were initially fractionated by column chromatography on Sephadex LH-20 with *n*-hexane, chloroform, and methanol (2:1:1) as the eluent. Subsequent purification involved silica gel column chromatography employing gradient mixtures of *n*-hexane and ethyl acetate of increasing polarity, followed by preparative HPTLC using an *n*-hexane-chloroform-acetone (6:3:1) solvent system.

**Figure 10:**
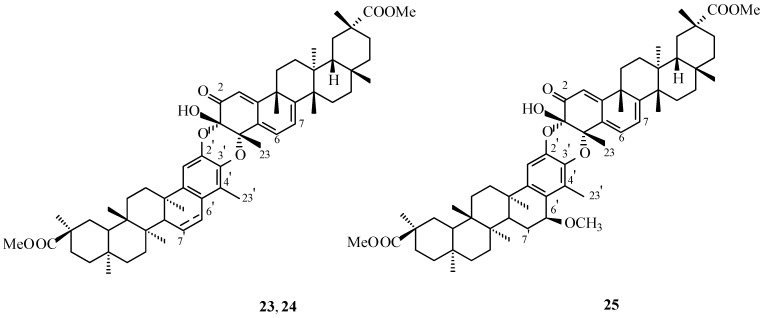
Structure of natural triterpene dimers **23**–**25**. **Legend**: **23**: scutionin αB; 6′,7′: double bond. **24**: 6′,7′-dihydroscutionin αB; 6′,7′: single bond. **25**: 6′-β-methoxydihydroscutionin αB.

In addition to the known triterpene dimers: scutionin αA (**8**, Figure 3), 7,8-dihydroscutionin αA (**9**, Figure 3), scutidin αA (**11**, Figure 3), 7,8-dihydroscutidin αB (**14**, Figure 3), cangorosin A (**17**, Figure 7), 6′,7′-dihydroisocangorosin A (**19**, Figure 7), and xuxuarine Eβ (**20**, Figure 8), all previously reported from *M. blepharodes*, three previously undescribed dimeric triterpenes (**23**–**25**, Figure 10) were isolated from *M. magellanica*.

The first of the newly identified triterpene dimers, scutionin αB (**23**, Figure 10), was isolated as a pale yellow, amorphous solid (Table 1). Its FAB-MS spectrum displayed a molecular ion at *m*/*z* 929, consistent with the molecular formula C_60_H_80_O_8_. ^1^H and ^13^C NMR analyses confirmed that compound **23** consists of two pristimerin-type triterpene units, one in a quinoid form (T_a_) and the other in an aromatic form (T_b_). Overall, its NMR data closely resembled those of xuxuarine Eα (**22**, Figure 9), with notable differences localized in the B ring of the T_b_ unit.

In the ^1^H NMR spectrum, two double doublets at δ 6.67 and 5.91 ppm (Table 2) were assigned to the olefinic protons at the C-6′ and the C-7′ atoms, forming a double bond conjugated with the aromatic A ring. The absence of a carbonyl function at the C-6 atom of the T_a_ unit was supported by the lack of an IR absorption band near 1677 cm^−1^. Signals at δ 6.06, 6.21, and 5.94 ppm were attributed to vinyl protons at the C-1, the C-6, and the C-7 positions (Table 2), characteristic of a quinoid triterpene system. In the ^13^C NMR spectrum, resonances at δ 91.8 and 79.1 ppm, assigned to the C-3 and the C-4 atoms (Table 3), respectively, indicated a cis-oriented 3,4-dioxy linkage. The absolute configuration at these centers was determined to be 3S,4S. Structural analysis established [3-*O*-2′] and [4-*O*-3′] ether linkages between the A rings of the T_a_ and T_b_ units, confirming scutionin αB (**23**, Figure 10) as a regioisomer of scutionin αA (**8**, Figure 3) [37].

The second triterpene dimer obtained from the *M. magellanica* extract, designated compound **24** (Figure 10), was also isolated as a pale yellow, amorphous solid (Table 1). Comparative analysis of its ^1^H and ^13^C NMR spectra with those of scutionin αB (**23**, Figure 10) revealed the principal distinction to be the absence of resonances at δ 6.67 and 5.91 ppm in the ^1^H NMR spectrum (Table 2), as well as signals at δ 124.0 and 129.4 ppm in the ^13^C NMR spectrum (Table 3). These missing signals correspond to the conjugation of the C-6′–C-7′ double bond present in the T_b_ unit of compound **23**, indicating saturation at this position in dimer **24**.

ROESY correlations, together with CD data, supported a cis orientation across the 3,4-dioxy linkage. Structural analysis further established that the A rings of the T_a_ and T_b_ units are connected through [3-*O*-2′] and [4-*O*-3′] ether bridges. On the basis of the combined spectroscopic evidence, compound **24** (Figure 10) was identified as 6′,7′-dihydroscutionin αB [37].

The third dimeric triterpene isolated from the *M. magellanica* extract, designated compound **25** (Figure 10), was also identified as a pristimerin-pristimerin-type dimer based on spectroscopic evidence [37]. Comparison of its ^1^H and ^13^C NMR spectra with those of compound **24** (Figure 10) revealed two additional signals at δ 4.28 and 3.38 ppm in the ^1^H NMR spectrum (Table 2) and at δ 72.5 and 55.4 ppm in the ^13^C NMR spectrum (Table 2). These resonances were assigned to the proton at the C-6′ and to a methoxy group attached at the same position, respectively.

HMBC correlations, including three-bond interactions between the C-6′ proton and the C-4′ and the C-10′ carbons, as well as a two-bond coupling with the C-5′ atom, unambiguously located the methoxy substituent at the C-6′. ROESY data showing NOE interactions between the C-6′ methyl group and the C-6′ proton confirmed that the T_a_ and T_b_ units are linked via [3-*O*-2′] and [4-*O*-3′] ether bonds. The relative configuration of the methoxy group at the C-6′ was established as β based on NOE correlations. Circular dichroism analysis further indicated absolute configurations of 3S and 4S at the C-3 and the C-4 atoms of the T_a_ unit. On the basis of the combined spectroscopic data, compound **25** (Figure 10) was identified as 6′β-methoxydihydroscutionin.

The pristimerin-pristimerin-type triterpene dimers (**23**–**25**, Figure 10) were evaluated for antimicrobial activity against a panel of microorganisms comprising seven Gram-positive bacteria (*Staphylococcus aureus* ATCC 6538, *S. epidermidis* CECT 232, *S. saprophyticus* CECT 235, *Enterococcus faecalis* CECT 481, *Bacillus subtilis* CECT 39, *B. cereus* CECT 496, *Mycobacterium smegmatis* CECT 3032), four Gram-negative bacteria (*Escherichia coli* CECT 99, *Proteus mirabilis* CECT 170, *Salmonella* sp. CECT 456, *Pseudomonas aeruginosa* AK 958), and the yeast *Candida albicans* (UBC 1) (Table 4). None of the tested dimers exhibited antimicrobial activity, with minimum inhibitory concentrations exceeding 40 µg/mL.

In a second set of assays, compounds **23**–**25** (Figure 10) were assessed for cytotoxic effects against human cervical carcinoma (HeLa) and human laryngeal carcinoma (Hep-2) cell lines. All dimers were found to be inactive, displaying IC_50_ values above 40 µg/mL for HeLa cells and above 20 µg/mL for Hep-2 cells [37].

These findings support the hypothesis that molecular size plays a critical role in the biological activity of this class of compounds and suggest that such dimers may function as inactive storage forms in plants, capable of releasing biologically active monomeric units through a retro-Diels-Alder process.

## 10. *Maytenus chuchuhuasca* Pristimerin-Pristimerin-Type Triterpene Dimers—Advanced Structural Studies

In subsequent studies on triterpene dimers derived from a Brazilian medicinal plant of the *Celastraceae* family, Shirota and co-workers [38] identified the presence of additional minor dimeric constituents. The isolation and purification of these compounds proved challenging and required multiple chromatographic procedures, including repeated separations by ODS HPLC (Octadecylsilyl High-Performance Liquid Chromatography), ODS MPLC (Octadecylsilyl Medium-Pressure Liquid Chromatography), and conventional HPLC. Through these extensive efforts, a total of nine triterpene dimers were successfully isolated, among which two were conclusively identified as pristimerin-pristimerin-type dimers.

**Figure 11:**
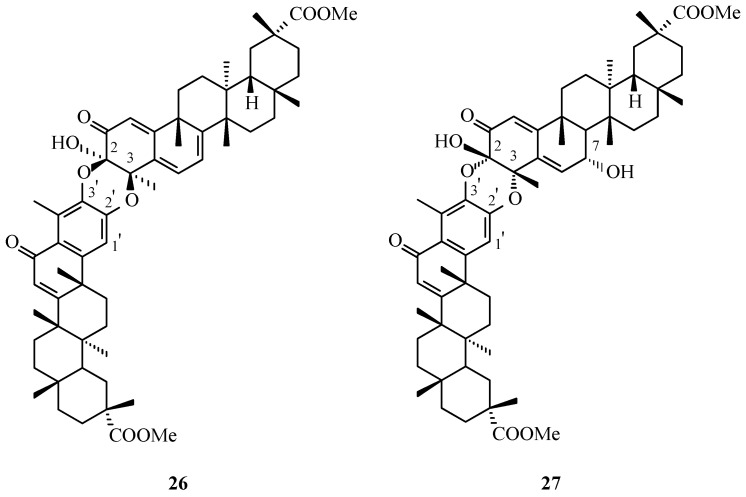
Structure of natural triterpene dimers **26**, **27**. **Legend**: **26**: isoxuxuarine Eβ. **27**: 7α-hydroxyisoxuxuarine Eα.

The first compound confirmed to possess a pristimerin-pristimerin framework was isolated as a pale yellow, amorphous solid (Table 1). Its FAB-MS spectrum displayed an [M+H]^+^ ion at *m*/*z* 943, and high-resolution FAB-MS analysis established the molecular formula as C_60_H_78_O_9_. Structural analysis demonstrated that one monomeric unit (T_a_) adopted a quinoid configuration, while the second unit (T_b_) was present in an aromatic form (**26**, Figure 11). The NMR spectroscopic features of dimer **26** closely resembled those of xuxuarine Eβ (**20**, Figure 8). Chemical shift values for the C-3, the C-4, and the C-23 atoms (δ 90.7, 77.2, and 24.2 ppm, respectively; Table 3), together with proton resonances assigned to the C-6 and the C-23′ atoms (δ 6.56 and 2.42 ppm, respectively; Table 2), supported an iso-type conjugation of the A and A′ rings through cis-oriented 3,4-dioxy linkages of the [2-*O*-3′] and [3-*O*-2′] types. These structural assignments were further corroborated by CD and ROESY (Rotating-frame Overhauser Effect Spectroscopy) analyses, leading to the designation of compound **26** (Figure 11) as isoxuxuarine Eβ.

The second pristimerin-pristimerin-type dimer, designated compound **27** (Figure 11), exhibited an [M+H]^+^ ion at *m*/*z* 960 in the FAB-MS spectrum. High-resolution FAB-MS analysis established its molecular formula as C_60_H_80_O_10_. ^1^H and ^13^C NMR spectroscopic data indicated hydroxylation of the conjugated ketone system in the quinoid triterpene unit (T_a_) at the C-7 position. Chemical shift values for the C-3, the C-4, and the C-23 atoms (δ 91.3, 79.1, and 22.4 ppm, respectively; Table 3), together with proton resonances at the C-6 and the C-23′ positions (δ 6.15 and 2.52 ppm, respectively; Table 2), supported an isoxuxuarine-type conjugation identical to that observed for compound **26** (Figure 11). This connectivity involved cis-oriented 3,4-dioxy linkages of the [2-*O*-3′] and [3-*O*-2′] types.

ROESY analysis revealed a key cross-peak between protons at the C-6 and the C-1′ positions confirming iso-type conjugation of the A and A′ rings. Additional NOE correlations between protons at the C-7 and the C-25 positions as well as between protons at the C-27 and the C-8 positions established the relative stereochemistry at the C-7 and the C-8 locations, indicating an α-oriented hydroxyl group at the C-7 and an α-oriented proton at the C-8 atom. Based on the combined spectroscopic evidence, compound **27** was identified as 7α-hydroxyisoxuxuarine Eα (Figure 11).

Jacobsen and co-workers [34] demonstrated that the reaction of pristimerin with DDQ induces a biomimetic coupling process, leading to the formation of xuxuarines Eα (**22**, Figure 9) and Eβ (**20**, Figure 8), along with an unusual monomeric derivative.

A solution of pristimerin (1.0 equiv) in dioxane was stirred with a slight excess of DDQ (1.2 equiv) at room temperature for 6 h, resulting in the formation of at least three products. Following workup, including dilution with ethyl acetate, extraction, drying, and solvent removal, repeated silica gel column chromatography afforded three major products.

Spectroscopic analysis (IR, ^1^H and ^13^C NMR, HR-FAB-MS, HMBC, NOE, and ROESY) confirmed that two of the isolated triterpene dimers were identical to xuxuarine Eα (**22**, Figure 9) and xuxuarine Eβ (**20**, Figure 8), previously obtained from natural sources by Gonzalez (**22**, Figure 9) [36] and Shirota (**20**, Figure 8) [33].

**Figure 12:**
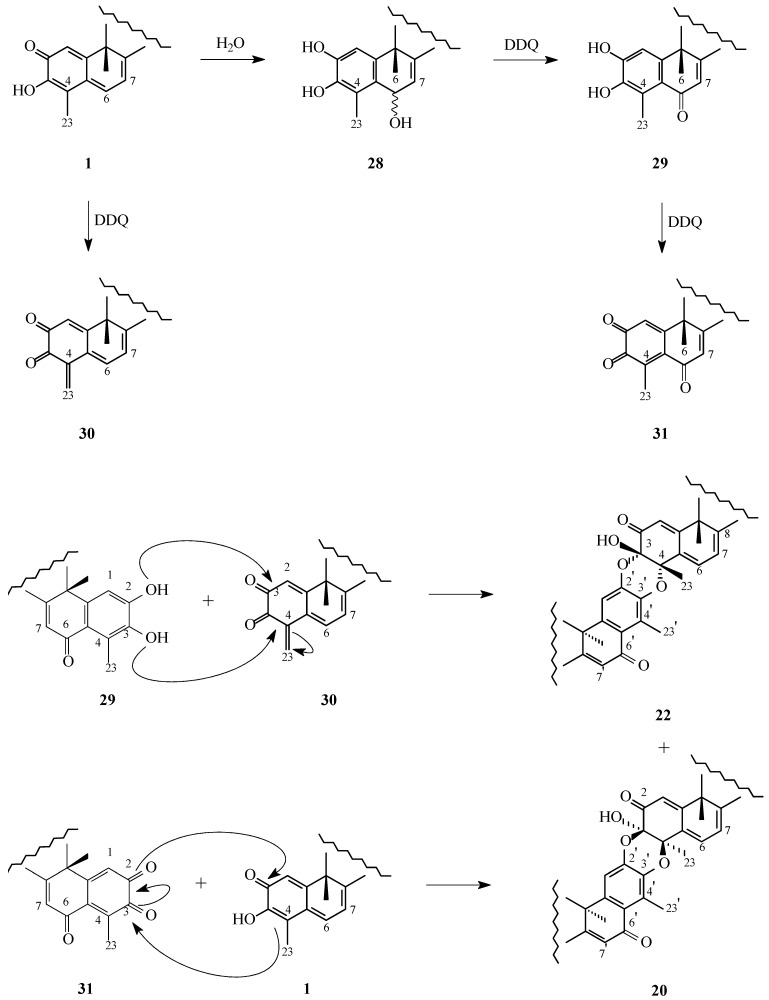
Possible reaction pathways for the formation of dimeric triterpenes: xuxuarine Eα (**22**) and Eβ (**20**) in oxidation reactions of pristimerin (**1**).

The formation of xuxuarines Eα (**22**, Figure 9) and Eβ (**20**, Figure 8) is proposed to involve initial conversion of the quinoid form of pristimerin (**1**, Figure 1), as presented in Figure 12, into alcohol **28** via hydration, followed by DDQ-mediated oxidation to yield 6-oxopristimerol (**29**, Figure 12). Subsequent coupling between quinoid pristimerin (**1**, Figure 1) and 6-oxopristimerol (**29**, Figure 12), leading to xuxuarines Eα (**22**, Figure 9) and Eβ (**20**, Figure 8), may proceed through two alternative pathways (Figure 12): (i) coupling of 6-oxopristimerol (**29**) with the oxidized form of pristimerin (**30**), or (ii) coupling of the oxidized form of 6-oxopristimerol (**31**) with quinoid pristimerin (**1**).

A plausible pathway for the formation of dimers **22** and **20** involves intermediate **30**, which bears an exo-methylene double bond conjugated with the quinone methide system. The presence of this exo-olefin significantly increases the reactivity of the molecule under oxidative conditions, making it a suitable intermediate for intermolecular coupling processes. Oxidation of pristimerin in the presence of DDQ can generate intermediate **30,** which may then undergo further activation to produce a highly reactive species capable of reacting with another triterpenoid unit present in the reaction mixture.

In this context, the exo-olefinic system of compound **30** can serve as the key reactive site for dimer formation. Nucleophilic attack by the oxygen functionality of a second monomeric unit followed by intramolecular trapping and subsequent oxidation could lead to the formation of the two ether bridges linking the A rings of the two triterpenoid fragments. This sequence would ultimately generate the dimeric framework observed in compounds **22** and **20**.

The formation of both products can be rationalized by alternative spatial approaches of the two reacting units during the coupling step, resulting in the generation of two stereochemically distinct dimers. These correspond to the diastereomeric structures identified as xuxuarines Eα (**22**) and Eβ (**7**).

## 11. *Maytenus orbiculatus* Closely Related Celastroloid Dimers

Triterpene dimers have also been reported from another member of the *Celastraceae* family, *Celastrus orbiculatus* (Thunb.) [39]. The root bark of *C. orbiculatus* was comminuted and extracted with an ethanol-water mixture (95:5, *v*/*v*). The resulting extract was partitioned between ethyl acetate and water, and the ethyl acetate-soluble fraction was subjected to successive chromatographic separations using different types of silica gel, such as H-60, ZCX-II, MCI gel CHP-20P, Sephadex LH-20 or HW-40, with various solvents or solvent mixtures as eluents. This procedure afforded ten previously unreported compounds, three of which were identified as triterpene dimers.

**Figure 13:**
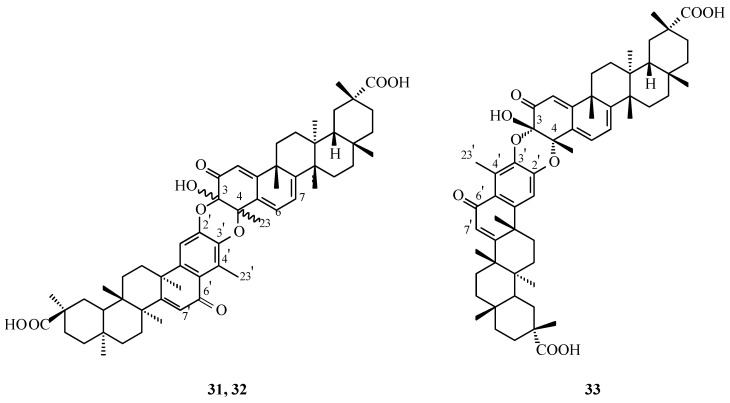
Structure of natural triterpene dimers **31**–**33**. **Legend**: **31**: celastroline Aα; cis 3,4-dioxy bond = α. **32**: celastroline Aβ; cis 3,4-dioxy bond = β. **33:** isocelastroline Aα.

The above-mentioned dimers (**31**–**33**, Figure 13) were shown to share the same molecular formula, C_58_H_74_O_9_, as established by HR-ESI-MS and ^13^C NMR analyses. The ^1^H and ^13^C NMR spectra of compounds **31**–**33** closely resembled those of xuxuarine Eα (**22**, Figure 9) and xuxuarine Eβ (**20**, Figure 8) synthesized from pristimerin (**1**, Figure 1) by Jacobsen [34], as well as those of isoxuxuarine Eα (≡scutidin αA, **11**, Figure 3) isolated from *M. chuchuhuasca* by Shirota [35], differing only in the absence of two methoxy signals in each case.

Because CD spectroscopy and NOESY correlations between the C-23′ methyl group and the C-6 proton allow differentiation between xuxuarine and isoxuxuarine isomers, while the chemical shifts of the C-3, the C-4, and the C-23 carbons are diagnostic for distinguishing α- and β-type dimers, detailed analysis of these spectroscopic features enabled unambiguous structural assignment of compounds **31**–**33** (Figure 13).

In CD spectra, α-type triterpene dimers typically exhibit a positive first Cotton effect at approximately 340 nm, whereas β-type dimers show a negative first Cotton effect near 390 nm. Accordingly, compounds **31** and **33** (Figure 13) displayed positive first Cotton effects (Δε = +14.80 and +18.61, respectively; Δε = molar extinction coefficient difference), while compound **32** (Figure 13) showed a negative first Cotton effect (Δε = −4.17). In the NOESY spectra, correlations between the C-6 proton and the C-23′ methyl protons were observed for dimers **31** and **32** (Figure 13), whereas compound **33** (Figure 13) exhibited correlations between the C-6 and C-1′ protons. Furthermore, in the ^13^C NMR spectra, chemical shifts of the C-3, the C-4, and the C-23 atoms, were observed at approximately δ 92, 77, and 22 ppm (Table 3), respectively for compounds **31** and **33** (Figure 13) [39]. These chemical shifts are characteristic of α-type dimers. The chemical shifts at approximately δ 91, 77, and 24 ppm (Table 3), as found for compound **32** (Figure 13) [39], are indicative of a β-type structure.

On the basis of the spectroscopic evidence discussed above, Wu and co-workers assigned the structures of triterpene dimers **31**–**33** as xuxuarine Eα-type, xuxuarine Eβ-type, and isoxuxuarine Eα-type, respectively [39]. Although compounds **31**–**33** are closely related to xuxuarine/isoxuxuarine dimers and are highly informative from a comparative spectroscopic perspective, their monomeric subunits were assigned as celastrol rather than pristimerin. They are therefore discussed here as closely related comparative celastroloids rather than as strict pristimerin-pristimerin dimers. Accordingly, compounds **31**–**33** were designated celastroline Aα (**31**), celastroline Aβ (**32**), and isocelastroline Aα (**33**).

## 12. Conclusions

**Figure 14:**
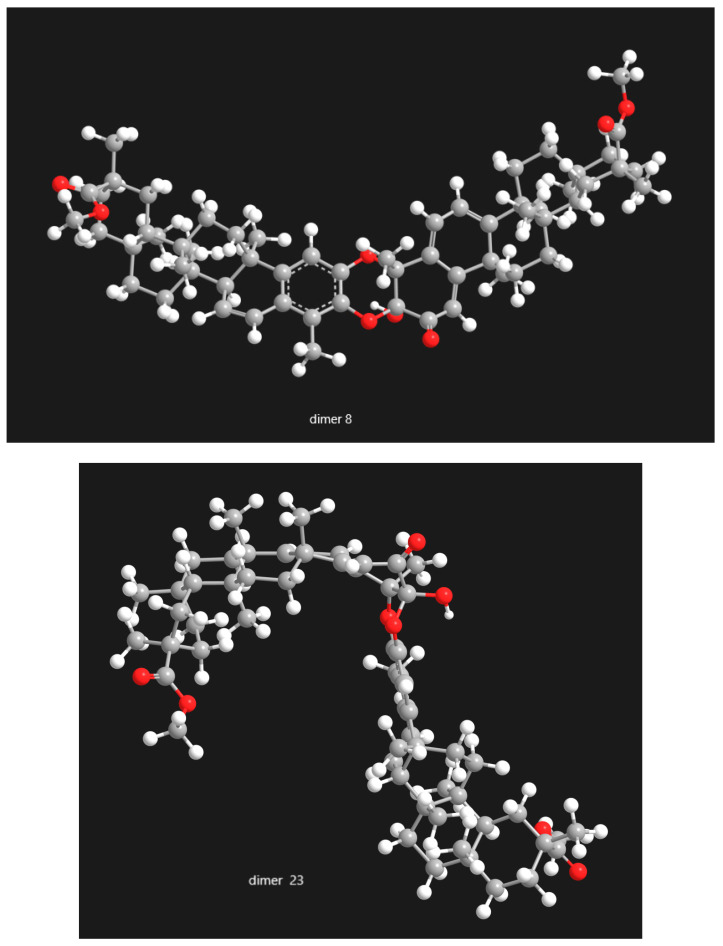
The selected categories of structural isomers of pristimerin-pristimerin-type dimmers: dimer A-type (dimer **8**) vs. dimer B-type (dimer **23**).

**Figure 15:**
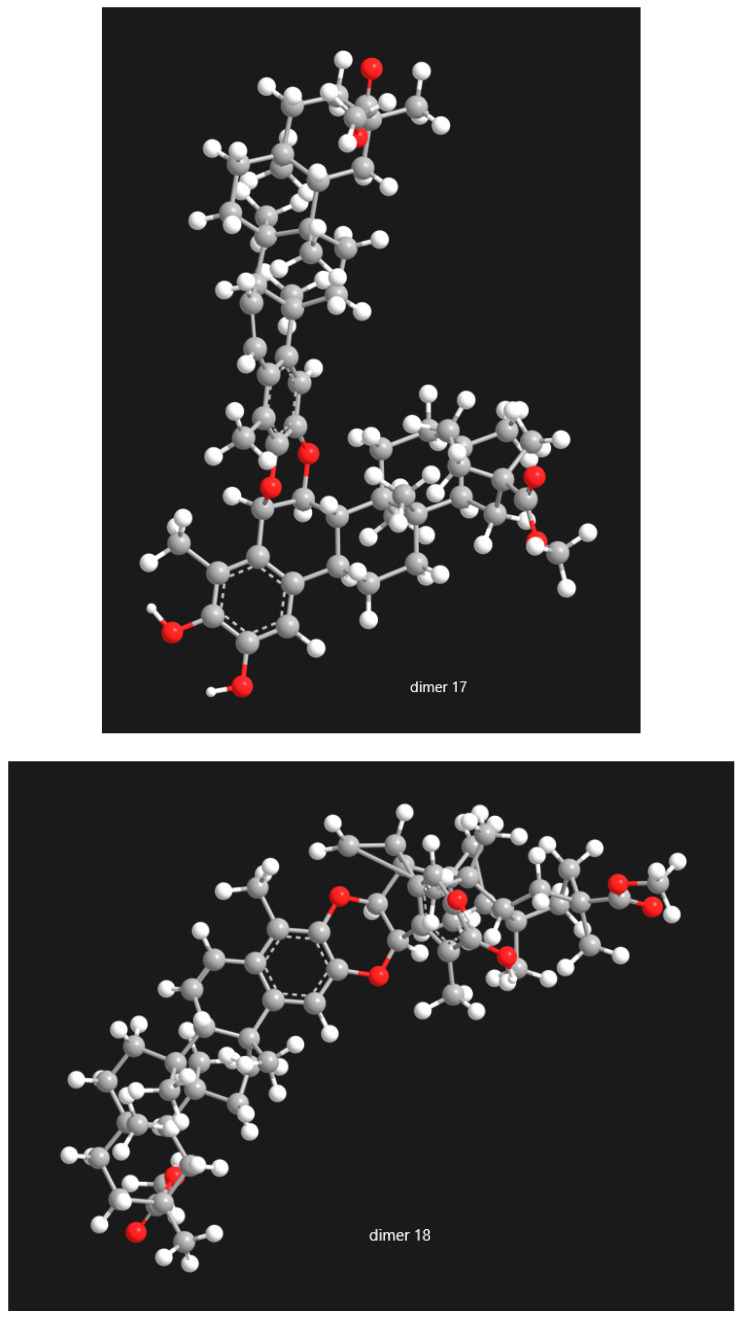
The selected categories of structural isomers of pristimerin-pristimerin-type dimmers: dimer type (dimer **17**) vs. isodimer type (dimer **18**).

**Figure 16:**
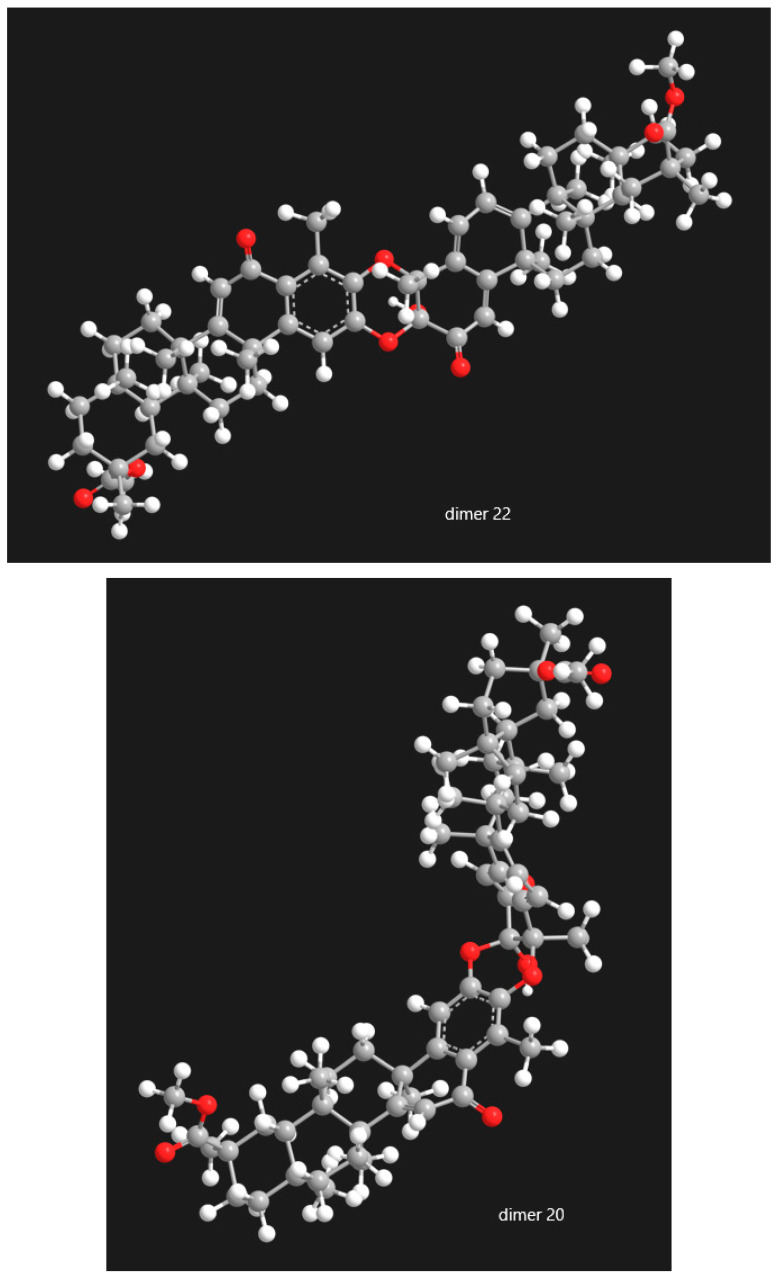
The selected categories of structural isomers of pristimerin-pristimerin-type dimmers: dimer α-type (dimer **22**) vs. dimer β-type (dimer **20**).

The study of pristimerin-pristimerin triterpene dimers reveals a structurally complex subclass of quinonemethide triterpenoids whose diversity arises from variations in oxidation state, ether-bridge connectivity, and stereochemistry. Research across *Maytenus* and *Celastrus* species has steadily expanded the known pool of such dimers while clarifying the biosynthetic principles underlying their formation. Evidence strongly supports dimerization via hetero-Diels-Alder reactions involving quinone or 2,3-diketone tautomers, generating numerous regioisomeric and stereoisomeric products, including α/β pairs, atropisomers, iso-type dimers, and other structural variants. In Figure 14, Figure 15 and Figure 16, the selected categories of structural isomers—including A/B-type dimers, dimer/isodimer forms, and α/β-type dimers—are presented in three-dimensional representations. These 3D models provide a clearer visualization of the stereochemical differences among the isomeric variants, highlighting variations in spatial arrangement, functional connectivity, and conformational geometry. By depicting the molecular architecture in this manner, Figure 14, Figure 15 and Figure 16 facilitates a more comprehensive understanding of how each isomer type differs in terms of its structural topology and potential physicochemical or biological properties.

The extensive structural investigations summarized in this review underscore the importance of advanced NMR techniques, such as 2D inverse-detection experiments, NOESY/ROESY correlations, and CD spectroscopy. These methodologies have resolved earlier ambiguities and enabled several structure revisions, particularly within the cangorosin and xuxuarine families, demonstrating the need for rigorous spectroscopic verification when studying highly oxygenated, conformationally flexible natural products. A further strength of this body of literature is that it documents several cases of structural reassignment and nomenclatural clarification, underscoring the need for caution when comparing older reports across this chemically complex subclass.

Available biological data indicate that pristimerin-pristimerin dimers generally display weak antibacterial and cytotoxic effects when compared with monomeric quinonemethide triterpenes. At present, these data do not support strong pharmacological conclusions but rather suggest that dimerization is associated primarily with structural diversification. The possibility that such dimers may serve as inactive or less-active reservoirs of monomeric triterpenoids remains an interesting working hypothesis, but it requires direct experimental verification.

Overall, pristimerin-derived dimers illustrate how small changes in oxidation or reaction trajectory can generate significant structural diversity within a single triterpenoid scaffold. They also highlight the utility of biomimetic oxidative coupling reactions—such as DDQ-mediated processes—in clarifying natural biosynthetic routes. Despite their limited pharmacological activity, these dimers offer valuable insight into reaction mechanisms, stereocontrol, and the chemical ecology of *Celastraceae* species. As such, they remain an important but underexplored group of natural products, meriting further mechanistic, biosynthetic, and ecological investigation to elucidate their roles in plant biology and natural product evolution.

## 13. Future Directions

Future research on pristimerin-pristimerin dimers should prioritize elucidating their biosynthetic origin and physiological relevance. Although many regio- and stereoisomers have been characterized, the enzymatic mechanisms governing quinone-diketone interconversion and ether-bridge formation remain unclear. Identifying oxidases, cyclases, and potential Diels-Alderase-like enzymes—supported by genomic and metabolomic analyses—will help determine whether these dimers are enzyme-mediated products or the result of spontaneous chemical coupling.

Progress is also expected from biomimetic and synthetic studies. Existing DDQ-mediated and model Diels-Alder reactions demonstrate that natural dimers can be reproduced and diversified in vitro. Targeted structural modifications, such as adjusting oxidation patterns or substituents, may yield analogues with improved physicochemical or biological properties, enabling deeper exploration of structure-activity relationships and clarifying the causes of the weak activity of native dimers.

Another essential direction is understanding the ecological function of these compounds. The hypothesis that pristimerin-based dimers act as inactive storage or transport forms requires verification through studies of retro-Diels-Alder reactivity under physiological conditions and experiments examining plant stress responses that may trigger dimer cleavage. Their potential roles in defense signaling or other ecological interactions should also be explored.

Finally, expanded pharmacological screening beyond conventional antibacterial and cytotoxic assays may reveal subtle or context-dependent activities. Evaluating effects on redox regulation, immune pathways, or non-classical molecular targets could uncover functions not apparent in standard tests. Overall, advancing knowledge of these dimers will require coordinated efforts integrating biosynthesis, synthetic chemistry, chemical ecology, and broadened biological evaluation.

## Figures and Tables

**Table 1:** Sources and basic physical data of natural pristimerin-pristimerin-type triterpene dimers. **Legend**: **---** = data not given; * = concentration not given; **yell.** = yellow; **amorph.** = amorphous.

Dimer Name	Source	Appearance	m.p.	[α]_D_	Ref.
“Compound **5**” (**3**)	*Rzedowskia tolantonguensis* (roots)	---	---	---	[28]
	reaction of primerin with DDQ	---	---	−143° (CHCl_3_) *	
“Compound **6**” (**4**)	*Rzedowskia tolantonguensis* (roots)	---	---	---	
	reaction of primerin with DDQ	---	---	+260° (CHCl_3_) *	
cangorosin A (**5**)	*Maytenus ilicifolia* (no organ given)	colorless amorph. solid	---	+237.4° (*c* 0.25, CHCl_3_)	[29]
atropcangorosin A (**6**)		colorless powder	---	+76.4° (*c* 0.28, CHCl_3_)	
6′,7′-dihydroatropcangorosin A (**7**)		colorless powder	---	+87.3° (*c* 0.45, CHCl_3_)	
scutionin αA (**8**)	*Maytenus scutioides* (root bark)	yell. lacquer	---	−338.8 (*c* 0. 40, CHCl_3_)	[30]
7,8-dihydroscutionin αA (**9**)		yell. amorph. solid	186–187 °C	+254.7 (*c* 1.70, CHCl_3_)	
7,8-dihydroscutionin βA (**10**)		yell. lacquer	---	−144.0 (*c* 0.15, CHCl_3_)	
scutidin αA (**11**)		yell. amorph. solid	193–194 °C	+278.6 (*c* 4.10, CHCl_3_)	
scutidin αA (**11**) (= isoxuxuarine Eα)	*Maytenus chuchuhuasca* (bark)	yell. amorph. solid	208–212 °C	+566.8 (*c* 0.41, CHCl_3_)	[31]
7,8-dihydroscutionin αB (**12**)	*Maytenus scutioides* (root bark)	yell. lacquer	---	+208.9 (*c* 0.80, CHCl_3_)	[30]
7,8-dihydroscutionin βB (**13**)			---	−69.8 (*c* 0.50, CHCl_3_)	
7,8-dihydroscutidin αB (**14**)		yell. crystalline solid	81–82 °C	+3.33 (*c* 0.2, CHCl_3_)	
compound **16**	reaction of 4α-hydroxypristimerin with pristimerin	pale yellow lacquer	---	---	
cangorosin A, with revised structure (**17**)	*Maytenus ilicifolia* (root bark)	colorless amorph. solid	---	+237.4° (*c* 0.25, CHCl_3_)	[32]
isocangorosin A (**18**) = atropcangorosin A according to [29]		colorless amorph. solid	---	+76.4° (*c* 0.28, CHCl_3_)	
6′,7′-dihydroisocangorosin A (**19**) = 6′,7′-dihydroatropcangorosin A (**7**) according to [29]		colorless amorph. solid	---	+87.3° (*c* 0.45, CHCl_3_)	
xuxuarine Eβ (**20**)	*Maytenus chuchuhuasca* (bark)	yell. amorphous solid	---	−352.9 (*c* 0.14, CHCl_3_)	[33]
	reaction of pristimerin with DDQ	yell. powder	198–201 °C	−349.4 (c 1.0, CHCl_3_)	[34]
7,8-dihydroisoxuxuarine Eα (**21**)	*Maytenus chuchuhuasca* (bark)	pale yell. amorph. solid	---	+309.1 (*c* 0.56, CHCl_3_)	[35]
xuxuarine Eα (**22**)	*Maytenus blepharodes* (roots)	pale yell. amorph. solid	---	+352.2° (*c* 0.32, MeOH)	[36]
	reaction of pristimerin with DDQ	ell. powder	219–221 °C	+361.7 (*c* 1.0, CHCl_3_)	[34]
scutionin αB (**23**)	*Maytenus magellanica* (root bark)	pale yell. amorph. solid	---	+282.3° (*c* 1.58, CHCl_3_)	[37]
6′,7′-dihydroscutionin αB (**24**)		pale yell. amorph. solid	---	+311.4° (*c* 0.22, CHCl_3_)	
6′β-methoxy-6′,7′-dihydroscutionin αB (**25**)		pale yell. amorph. solid	---	+276.2° (*c* 0.21, CHCl_3_)	
isoxuxuarine Eβ (**26**)	*Maytenus chuchuhuasca* (bark)	yell. amorph. solid	---	---	[38]
7α-hydroxyisoxuxuarine Eα (**27**)		yell. amorph. solid	---	---	
celastroline Aα (**31**)	*Celastrus orbiculatus* (root bark)	yell. amorph. solid	---	+488° (*c* 0.088, acetone)	[39]
celastroline Aβ (**32**)		yell. amorph. solid	---	−366° (*c* 0.075, acetone)	
isocelastroline Aα (**33**)		yell. amorph. solid	---	+587° (*c* 0.06, acetone)	

**Table 4:** Pharmacological activity of natural triterpene dimers.

Dimer Name	Activity	Results	Ref.
scutionin αA (**8**)	Antibacterial (ATCC 6538)	CMI > 20 µg/mL	[30]
7,8-dihydroscutionin αA (**9**)		CMI > 20 µg/mL	
7,8-dihydroscutionin βA (**10**)		CMI > 20 µg/mL	
scutidin αA (**11**)		CMI > 20 µg/mL	
7,8-dihydroscutionin αB (**12**)		CMI > 20 µg/mL	
7,8-dihydroscutionin βB (**13**)		CMI > 20 µg/mL	
7,8-dihydroscutidin αB (**14**)		CMI > 20 µg/mL	
scutionin αA (**8**)	Antibacterial (CECT 232)	CMI > 20 µg/mL	[30]
7,8-dihydroscutionin αA (**9**)		CMI > 20 µg/mL	
7,8-dihydroscutionin βA (**10**)		CMI > 20 µg/mL	
scutidin αA (**11**)		CMI > 20 µg/mL	
7,8-dihydroscutionin αB (**12**)		CMI > 20 µg/mL	
7,8-dihydroscutionin βB (**13**)		CMI > 20 µg/mL	
7,8-dihydroscutidin αB (**14**)		CMI > 20 µg/mL	
scutionin αA (**8**)	Antibacterial (CECT 235)	CMI > 20 µg/mL	[30]
7,8-dihydroscutionin αA (**9**)		CMI > 20 µg/mL	
7,8-dihydroscutionin βA (**10**)		CMI > 20 µg/mL	
scutidin αA (**11**)		CMI > 20 µg/mL	
7,8-dihydroscutionin αB (**12**)		CMI > 20 µg/mL	
7,8-dihydroscutionin βB (**13**)		CMI > 20 µg/mL	
7,8-dihydroscutidin αB (**14**)		CMI > 20 µg/mL	
scutionin αA (**8**)	Antibacterial (CECT 39)	CMI > 20 µg/mL	[30]
7,8-dihydroscutionin αA (**9**)		CMI > 20 µg/mL	
7,8-dihydroscutionin βA (**10**)		CMI > 20 µg/mL	
scutidin αA (**11**)		CMI > 20 µg/mL	
7,8-dihydroscutionin αB (**12**)		CMI > 20 µg/mL	
7,8-dihydroscutionin βB (**13**)		CMI > 20 µg/mL	
7,8-dihydroscutidin αB (**14**)		CMI > 20 µg/mL	
scutionin αA (**8**)	Antibacterial (CECT 29)	CMI > 20 µg/mL	[30]
7,8-dihydroscutionin αA (**9**)		CMI > 20 µg/mL	
7,8-dihydroscutionin βA (**10**)		CMI > 20 µg/mL	
scutidin αA (**11**)		CMI > 20 µg/mL	
7,8-dihydroscutionin αB (**12**)		CMI > 20 µg/mL	
7,8-dihydroscutionin βB (**13**)		CMI > 20 µg/mL	
7,8-dihydroscutidin αB (**14**)		CMI > 20 µg/mL	
scutionin αA (**8**)	Antibacterial (CECT 99)	CMI > 20 µg/mL	[30]
7,8-dihydroscutionin αA (**9**)		CMI > 20 µg/mL	
7,8-dihydroscutionin βA (**10**)		CMI > 20 µg/mL	
scutidin αA (**11**)		CMI > 20 µg/mL	
7,8-dihydroscutionin αB (**12**)		CMI > 20 µg/mL	
7,8-dihydroscutionin βB (**13**)		CMI > 20 µg/mL	
7,8-dihydroscutidin αB (**14**)		CMI > 20 µg/mL	
scutionin αA (**8**)	Antibacterial (CECT 170)	CMI > 20 µg/mL	[30]
7,8-dihydroscutionin αA (**9**)		CMI > 20 µg/mL	
7,8-dihydroscutionin βA (**10**)		CMI > 20 µg/mL	
scutidin αA (**11**)		CMI > 20 µg/mL	
7,8-dihydroscutionin αB (**12**)		CMI > 20 µg/mL	
7,8-dihydroscutionin βB (**13**)		CMI > 20 µg/mL	
7,8-dihydroscutidin αB (**14**)		CMI > 20 µg/mL	
scutionin αA (**8**)	Antibacterial (UBC 2)	CMI > 20 µg/mL	[30]
7,8-dihydroscutionin αA (**9**)		CMI > 20 µg/mL	
7,8-dihydroscutionin βA (**10**)		CMI > 20 µg/mL	
scutidin αA (**11**)		CMI > 20 µg/mL	
7,8-dihydroscutionin αB (**12**)		CMI > 20 µg/mL	
7,8-dihydroscutionin βB (**13**)		CMI > 20 µg/mL	
7,8-dihydroscutidin αB (**14**)		CMI > 20 µg/mL	
scutionin αA (**8**)	Antibacterial (AK 958)	CMI > 20 µg/mL	[30]
7,8-dihydroscutionin αA (**9**)		CMI > 20 µg/mL	
7,8-dihydroscutionin βA (**10**)		CMI > 20 µg/mL	
scutidin αA (**11**)		CMI > 20 µg/mL	
7,8-dihydroscutionin αB (**12**)		CMI > 20 µg/mL	
7,8-dihydroscutionin βB (**13**)		CMI > 20 µg/mL	
7,8-dihydroscutidin αB (**14**)		CMI > 20 µg/mL	
scutionin αA (**8**)	Cytotoxic (HeLa)	not active	[30]
7,8-dihydroscutionin αA (**9**)		not active	
7,8-dihydroscutionin βA (**10**)		not active	
scutidin αA (**11**)		not active	
7,8-dihydroscutionin αB (**12**)		not active	
7,8-dihydroscutionin βB (**13**)		not active	
7,8-dihydroscutidin αB (**14**)		not active	
scutionin αA (**8**)	Cytotoxic (Hep-2)	not active	[30]
7,8-dihydroscutionin αA (**9**)		not active	
7,8-dihydroscutionin βA (**10**)		not active	
scutidin αA (**11**)		not active	
7,8-dihydroscutionin αB (**12**)		not active	
7,8-dihydroscutionin βB (**13**)		not active	
7,8-dihydroscutidin αB (**14**)		not active	
scutionin αB (**23**)	Antibacterial (ATCC 6538)	CMI up to 40 µg/mL	[37]
6′,7′-dihydroscutionin αB (**24**)		CMI up to 40 µg/mL	
6′β-methoxydihydroscutionin αB (**25**)		CMI up to 40 µg/mL	
scutionin αB (**23**)	Antibacterial (CECT 232)	CMI up to 40 µg/mL	[37]
6′,7′-dihydroscutionin αB (**24**)		CMI up to 40 µg/mL	
6′β-methoxydihydroscutionin αB (**25**)		CMI up to 40 µg/mL	
scutionin αB (**23**)	Antibacterial (CECT 235)	CMI up to 40 µg/mL	[37]
6′,7′-dihydroscutionin αB (**24**)		CMI up to 40 µg/mL	
6′β-methoxydihydroscutionin αB (**25**)		CMI up to 40 µg/mL	
scutionin αB (**23**)	Antibacterial (CECT 481)	CMI up to 40 µg/mL	[37]
6′,7′-dihydroscutionin αB (**24**)		CMI up to 40 µg/mL	
6′β-methoxydihydroscutionin αB (**25**)		CMI up to 40 µg/mL	
scutionin αB (**23**)	Antibacterial (CECT 39)	CMI up to 40 µg/mL	[37]
6′,7′-dihydroscutionin αB (**24**)		CMI up to 40 µg/mL	
6′β-methoxydihydroscutionin αB (**25**)		CMI up to 40 µg/mL	
scutionin αB (**23**)	Antibacterial (CECT 496)	CMI up to 40 µg/mL	[37]
6′,7′-dihydroscutionin αB (**24**)		CMI up to 40 µg/mL	
6′β-methoxydihydroscutionin αB (**25**)		CMI up to 40 µg/mL	
scutionin αB (**23**)	Antibacterial (CECT 3032)	CMI up to 40 µg/mL	[37]
6′,7′-dihydroscutionin αB (**24**)		CMI up to 40 µg/mL	[37]
6′β-methoxydihydroscutionin αB (**25**)		CMI up to 40 µg/mL	[37]
scutionin αB (**23**)	Antibacterial (CECT 99)	CMI up to 40 µg/mL	[37]
6′,7′-dihydroscutionin αB (**24**)		CMI up to 40 µg/mL	[37]
6′β-methoxydihydroscutionin αB (**25**)		CMI up to 40 µg/mL	[37]
scutionin αB (**23**)	Antibacterial (CECT 170)	CMI up to 40 µg/mL	[37]
6′,7′-dihydroscutionin αB (**24**)		CMI up to 40 µg/mL	[37]
6′β-methoxydihydroscutionin αB (**25**)		CMI up to 40 µg/mL	[37]
scutionin αB (**23**)	Antibacterial (CECT 456)	CMI up to 40 µg/mL	[37]
6′,7′-dihydroscutionin αB (**24**)		CMI up to 40 µg/mL	[37]
6′β-methoxydihydroscutionin αB (**25**)		CMI up to 40 µg/mL	[37]
scutionin αB (**23**)	Antibacterial (AK 958)	CMI up to 40 µg/mL	[37]
6′,7′-dihydroscutionin αB (**24**)		CMI up to 40 µg/mL	[37]
6′β-methoxydihydroscutionin αB (**25**)		CMI up to 40 µg/mL	[37]
scutionin αB (**23**)	Antifungal (UBC 1)	CMI up to 40 µg/mL	[37]
6′,7′-dihydroscutionin αB (**24**)		CMI up to 40 µg/mL	[37]
6′β-methoxydihydroscutionin αB (**25**)		CMI up to 40 µg/mL	[37]
scutionin αB (**23**)	Cytotoxic (HeLa)	IC_50_ up to 40 µg/mL	[37]
6′,7′-dihydroscutionin αB (**24**)		IC_50_ up to 40 µg/mL	[37]
6′β-methoxydihydroscutionin αB (**25**)		IC_50_ up to 40 µg/mL	[37]
scutionin αB (**23**)	Cytotoxic (Hep-2)	IC_50_ up to 20 µg/mL	[37]
6′,7′-dihydroscutionin αB (**24**)		IC_50_ up to 20 µg/mL	
6′β-methoxydihydroscutionin αB (**25**)		IC_50_ up to 20 µg/mL	

## Data Availability

All data supporting the findings of this study are contained within the article. No additional data were generated or are available.

## Abbreviations

The following abbreviations are used in this manuscript (abbreviations are listed in alphabetical order):

[α]_D_	specific rotation
β-CH_3_	methyl group at the β conformation
δ	chemical shift
Δε	molar extinction coefficient difference
μg/mL	micrograms per milliliter, 10^−6^ g per 10^−3^ L
ν	stretching frequency
^1^H NMR	Proton Nuclear Magnetic Resonance
^13^C NMR	Carbon Nuclear Magnetic Resonance
3-*O*-3′, etc.	ether linkage between the C-3 of one triterpene unit and the C-3′ of the second triterpene unit
3S,4S, etc.	carbon atom number 3 in S configuration and carbon atom number 4 in S configuration according to the Cahn-Ingold-Prelog Rule
ADMET	absorption, distribution, metabolism, excretion and toxicity parameters
AK 958	bacterial strain: *Pseudomonas aeruginosa*
amorph.	amorphous
ATCC 6538	bacterial strain: *Staphylococcus aureus*
br/s	broad singlet
*c*	concentration
C-3, C-12, etc.	number of C atom within the molecule of a compound
CECT 170	bacterial strain: *Proteus mirabilis*
CECT 232	bacterial strain: *Staphylococcus epidermidis*
CECT 235	bacterial strain: *Staphylococcus saprophyticus*
CECT 29	bacterial strain: *Bacillus pumilus*
CECT 3032	bacterial strain: *Mycobacterium smegmatis*
CECT 39	bacterial strain: *Bacillus subtilis*
CECT 456	bacterial strain: *Salmonella* sp.
CECT 481	bacterial strain: *Enterococcus faecalis*
CECT 496	bacterial strain: *Bacillus cereus*
CECT 99	bacterial strain: *Escherichia coli*
CH_2_Cl_2_	methylene chloride
CHCl_3_	chloroform
CD	circular dichroism
cm^−1^	reciprocal centimeter (or inverse centimeter); a unit of wavenumber
d	doublet
dd	doublet of doublets
ddd	doublet of doublets of doublets
DDQ	2,3-dichloro-5,6-dicyanobenzoquinone
DMDO	dimethyldioxirane
DMF	N,N-dimethylformamide
FAB-MS	Fast Atom Bombardment Mass Spectrometry
H-60, ZCX-II, etc.	different types of silica gel in chromatography
HeLa	cancer cell line: human cervical carcinoma
Hep-2	cancer cell line: human laryngeal carcinoma
HMBC	Heteronuclear Multiple Bond Correlation
HMQC	Heteronuclear Multiple-Quantum Correlation or Heteronuclear Multiple-Quantum Coherence
HPTLC	High-Performance Thin-Layer Chromatography
HR-FAB-MS	High-Resolution Fast Atom Bombardment Mass Spectrometry
Hz	hertz (the unit of frequency)
IR	Infrared
*J*	coupling constant
JNK	c-Jun N-terminal kinase
[M+H]^+^	protonated molecular ion in mass spectrometry
m	multiplet
*m*/*z*	mass-to-charge ratio of ions in mass spectrometry
MAPK	Mitogen-Activated Protein Kinase
m.p.	melting point
NF-κB	nuclear factor kappa B
NMR	Nuclear Magnetic Resonance
NOE	Nuclear Overhauser Effect
NOESY	Nuclear Overhauser Effect Spectroscopy
ODS HPLC	Octadecylsilyl High-Performance Liquid Chromatography
ODS MPLC	Octadecylsilyl Medium-Pressure Liquid Chromatography
PI3K/Akt	phosphatidylinositol 3-kinase/protein kinase B signaling pathway
ppm	part per million, 1 × 10^−6^
Ref.	Reference
ROESY	Rotating-frame Overhauser Effect Spectroscopy
ROR	Research Organisation Registry
s	singlet
SAR	structure-activity relationship
STAT3	signal transducer and activator of transcription 3
T_a_, T_b_	triterpene “a” unit, triterpene “b” unit
UBC 1	bacterial strain: *Candida albicans*
UBC 2	bacterial strain: *Salmonella typhimurium*
*v*/*v*	volume per volume, volumes ratio
yell.	yellow
